# A second hybrid-binding domain modulates the activity of *Drosophila* ribonuclease H1

**DOI:** 10.1093/jb/mvaa067

**Published:** 2020-07-02

**Authors:** Jose M González de Cózar, Maria Carretero-Junquera, Grzegorz L Ciesielski, Sini M Miettinen, Markku Varjosalo, Laurie S Kaguni, Eric Dufour, Howard T Jacobs

**Affiliations:** m1 Faculty of Medicine and Health Technology, FI-33014 Tampere University, Finland; m2 Department of Biochemistry and Molecular Biology, Michigan State University, East Lansing, MI 48824, USA; m3 Department of Chemistry, Auburn University at Montgomery, Montgomery, AL 36117, USA; m4 Institute of Biotechnology, FI-00014 University of Helsinki, Finland

**Keywords:** biolayer interferometry, mitochondria, ribonuclease H, shotgun proteomics, single-stranded DNA-binding protein

## Abstract

In eukaryotes, ribonuclease H1 (RNase H1) is involved in the processing and removal of RNA/DNA hybrids in both nuclear and mitochondrial DNA. The enzyme comprises a C-terminal catalytic domain and an N-terminal hybrid-binding domain (HBD), separated by a linker of variable length, 115 amino acids in *Drosophila melanogaster* (*Dm*). Molecular modelling predicted this extended linker to fold into a structure similar to the conserved HBD. Based on a deletion series, both the catalytic domain and the conserved HBD were required for high-affinity binding to heteroduplex substrates, while loss of the novel HBD led to an ∼90% drop in *K*_cat_ with a decreased *K*_M_, and a large increase in the stability of the RNA/DNA hybrid-enzyme complex, supporting a bipartite-binding model in which the second HBD facilitates processivity. Shotgun proteomics following *in vivo* cross-linking identified single-stranded DNA-binding proteins from both nuclear and mitochondrial compartments, respectively RpA-70 and mtSSB, as prominent interaction partners of *Dm* RNase H1. However, we were not able to document direct and stable interactions with mtSSB when the proteins were co-overexpressed in S2 cells, and functional interactions between them *in vitro* were minor.

## Graphical Abstract

**Figure:**
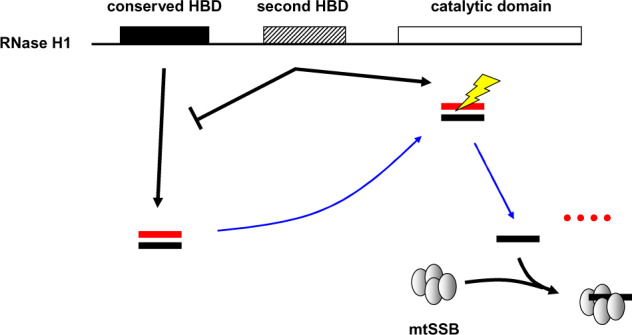

In eukaryotes, ribonuclease H1 (RNase H1) is present both in mitochondria and in the nucleus, as distinct translation products encoded by a single mRNA (1, 2). The enzyme digests the RNA strand of RNA/DNA heteroduplexes longer than 4 bp (3) and has been implicated in diverse DNA transactions in one or both cellular compartments, including DNA replication (2, 4–10), transcription (11–13), recombination (14, 15), repair (16, 17) and telomere maintenance (18, 19). The targeting of the enzyme to two cellular compartments complicates functional studies. Therefore, our understanding of its biology has instead relied heavily on biochemical analysis.

Eukaryotic RNase H1 presents a conserved domain organization (20). A nucleic acid-binding motif, denoted the hybrid-binding domain (HBD), which is also found in some bacteria such as *Bacillus halodurans* (21), is located near the N-terminus. The catalytic (RNase H) domain is found near the C-terminus, with an extended linker (or connection domain) located between these two domains. In human RNase H1, the HBD not only confers tight binding to RNA/DNA hybrid but also interacts weakly with dsRNA and even more weakly with dsDNA (22). Portions of its structure that interact with RNA and with DNA have been mapped precisely (22). However, the physiological or mechanistic role(s) of the HBD are not fully understood. It has been suggested to promote dimerization, conferring processivity to the enzyme (23) and interactions with other proteins (24). Since the first RNase H structure from bacteria was elucidated in 1990 (25, 26), a conserved three-dimensional organization has been observed in homologs of the enzyme from eukaryotes (27), prokaryotes (28, 29) and viruses (30–32). This specific structure involves four residues in close proximity, the DEDD motif (33), serving as the catalytic core of the enzyme (see Supplementary Fig. S1F). The catalytic domain of human RNase H1 has been studied extensively with respect to its binding affinity (34), cofactor requirements and structure (27). Among eukaryotes, the extended linker that connects the HBD and RNase H (catalytic) domains is the least phylogenetically conserved portion of the protein, both in length and primary sequence (Fig. 1) (35). The human linker spans 64 amino acids, whereas it is 48 residues long in *Caenorhabditis elegans* and 115 in *Drosophila melanogaster* (*Dm*). The current view is that the extended linker of the human enzyme confers flexibility to the RNase H domain whereas the HBD remains bound to the substrate (20); however, the variability of the linker among species remains unexplained.

**Fig. 1.**
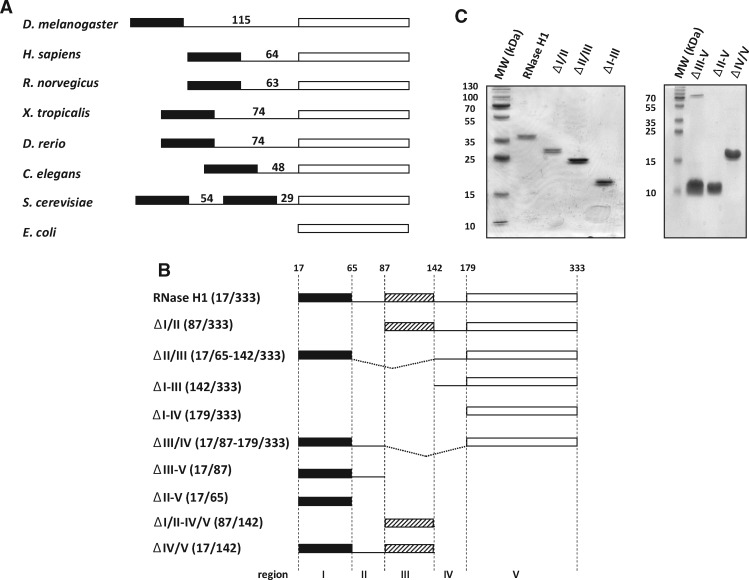
***Drosophila melanogaster* RNase H1 protein sequence organization and structure**. (**A**) Schematic representation of RNase H1 enzyme among different species (*Drosophila melanogaster*, *Homo sapiens*, *Rattus norvegicus*, *Xenopus tropicalis*, *Danio rerio*, *Caenorhabditis elegans*, *Saccharomyces cerevisiae* and *Escherichia coli*). Black box represents the conserved HBD and the empty box the catalytic domain. Length of the linker region (in amino acids) for each species is shown. Note that *S. cerevisiae* has two HBDs and two linkers (57). (**B**) Schematic representation of different *Dm* RNase H1 protein variants created in the study: RNase H1 (Rnh1, NCBI AAF59170.1 amino acids 17–333), ΔI/II (87–333), ΔII/III (17–65 + 142–333), ΔI–III (142–333), ΔI–IV (179-333), ΔIII/IV (17–87 + 179–333), ΔII–V (17–65), ΔIII–V (17–87), ΔI/II–IV/V (87–137) and ΔIV/V (17–142). The whole RNase H1 protein sequence has been divided into five regions (assumed to represent protein domains) bounded by amino acids 17, 65, 87, 142, 179 and 333. Black boxes represents the conserved HBD, the hatched boxes the second predicted HBD and the empty boxes the catalytic domain. (**C**) Purified proteins, as indicated, separated by SDS-PAGE and Coomassie staining, alongside molecular mass (MW) markers in kDa. Based on purity assessment (see Experimental Procedures) and overall protein concentrations, the effective amounts of each variant were estimated as follows: RNase H1 1.0 µg, ΔI/II 1.0 µg, ΔII/III 2.0 µg, ΔI–III 1.7 µg, ΔII–V 3.2 µg, ΔIII–V 4.8 µg and ΔIV/V 2.9 µg.

The *ribonuclease H1* (*rnh1*) gene is necessary for pupal development in *Dm* (36). *Rnaseh1* knockout mice also exhibit developmental lethality, with severely decreased copy number of mitochondrial DNA (mtDNA), suggesting an essential role of RNase H1 in mtDNA maintenance (5). Several point mutations in the human *RNASEH1* gene are associated with progressive external ophthalmoplegia (37–39), a syndromic disorder associated with mutations in various mtDNA maintenance proteins, such as the mitochondrial DNA polymerase gamma (Polγ) (40) or mitochondrial transcription factor A (TFAM) (41). Being targeted both to mitochondria and to the nucleus (1, 2), RNase H1 has been implicated in RNA/DNA turnover in both compartments (8, 42, 43). In mitochondria, heteroduplex turnover impacts mtDNA replication initiation and segregation (2, 7, 44) as well as mitochondrial RNA processing (2, 13, 45), while in the nucleus, it also affects DNA repair (16, 46) and telomere maintenance (18).

It remains unclear whether the lethality associated with *rnh1* deletion in *Drosophila* (36) is due to a requirement for its function in the nucleus, in mitochondria or both. Recently, we described the effects of *Drosophila rnh1* downregulation, which triggers decreased lifespan, mitochondrial dysfunction and mtDNA depletion (2). This phenotype was associated with abnormalities in mtDNA replication, notably at the origin and in regions where the transcription machinery progresses in the opposite direction to that of DNA replication (2).

In the present study, in view of the preliminary findings of previous investigators (47), we set out to determine the biochemical properties of the different domains of *Dm* RNase H1, notably that of the extended linker, via functional studies of a deletion series *in vitro*, and by screening for interacting proteins. This leads us to propose a new conceptual mechanism facilitating the processivity of the enzyme, as well as an interaction with mitochondrial single-stranded DNA-binding protein (mtSSB) that appears to be robust but indirect.

## Materials and Methods

### Molecular modelling

*Dm* RNase H1 protein structure was modelled by SWISS-MODEL (Biozentrum, University of Basel, Switzerland) (48–50). Its amino acid sequence was first compared with those of proteins from the protein data bank (PDB) that have crystallographically determined structures. Potential templates were then ranked, based on estimated GMQE (global model quality estimation). Model quality was assessed by QMEAN, a value calculated by global and local geometrical characteristics of the model with respect to the template. Models were analysed and visualized by PyMol (Schrödinger).

### Cloning into expression vectors

*rnh1* and *mtSSB* cDNAs were derived by PCR using methods described previously (2) and primers as listed in Supplementary Table S1. The *rnh1* cDNA was cloned into pET-26b(+) (Novagen, Merck Millipore) for bacterial expression with an in-frame C-terminal  6x-His tag. Partially deleted *rnh1* variants (Fig. 1B) were generated by a two-step PCR procedure, as described previously (2). PCR-based site-directed mutagenesis was used to create variant cDNAs bearing the D252N point mutation, predicted to abolish nuclease activity in RNase H1. For expression in *Drosophila* S2 cells (51), the *mtSSB* coding sequence was cloned into pMT-puro (Addgene) using a two-step procedure. It was first amplified and inserted into pMT/V5-His B (ThermoFisher Scientific), using primers that introduced EcoRI and XhoI restriction sites, then recloned into pMT-puro using primers that added KpnI and PmeI restriction sites, also eliminating the V5-His tag but adding a C-terminal HA [(amino acids 98-106) derived from human influenza hemagglutinin] tag in its place (see Supplementary Table S1). Successfully transfected colonies were selected by plating on 0.5 μg/ml puromycin (InvivoGen). All plasmids were sequenced before use.

### Protein expression and purification

Competent BL21 Star^TM^ (DE3) *Escherichia* *coli* cells (ThermoFisher Scientific) were transformed with pET-26b(+)-derived DNA constructs using heat shock and selected on 50 μg/ml kanamycin plates. Cells from single colonies were grown overnight in 200 ml L-broth (LB, 1% tryptone, 1% NaCl, 0.5% yeast extract, all w/v), supplemented with 50 μg/ml kanamycin, at 37°C with shaking at 250 rpm. The culture was then diluted into 4 l of LB (in eight 2-l Erlenmeyer flasks) and incubated at 37°C with shaking until it reached an OD_600_ of ∼0.7. Protein expression was induced by the addition of IPTG (isopropyl β-d-1-thiogalactopyranoside) to 400 µM and a further incubation for 3 h. Cells were harvested by 10-min centrifugation at 5,000 *g*_max_ and stored at −80 C°. Bacterial pellets were thawed in ice-cold resuspension buffer (30 mM Tris-HCl, 200 mM KCl, 2 mM DTT, 10% glycerol, pH 8.0) containing, per 25 ml, one cOmplete^TM^ EDTA-free Protease Inhibitor Cocktail tablet (Roche). Lysozyme (Invitrogen) was added to 200 μg/ml on ice for 40 min, after which cells were lysed by three rounds of sonication on ice, using a Vibra Cell (VC) 505 sonicator (Sonic & Materials, Inc.), fitted with a 13-mm probe, set to 60% amplitude with a 1 s/2 s on/off cycle for 3 min. Where needed, a fourth round of sonication was added. The lysate was ultracentrifuged at 100,000 *g*_max_ for 30 min at 4°C, after which the supernatant was filtered through a 0.45-µm nylon syringe filter (GE Healthcare Whatman™ Uniflo) and loaded dropwise overnight onto a 3-ml Ni-NTA agarose (ThermoFisher Scientific) column (Qiagen 30230) pre-washed in water then pre-equilibrated with equilibration buffer (30 mM Tris-HCl, 200 mM KCl, 2 mM DTT, 10% glycerol, 5 mM imidazole, pH 8.0, plus the protease inhibitor). All chromatography and gel filtration steps were conducted at 4°C. Non-specifically bound proteins were removed by successively washing with 15 ml buffer 1 (30 mM Tris-HCl, 200 mM KCl, 2 mM DTT, 10% glycerol, 25 mM imidazole, pH 8.0, plus the protease inhibitor) and 15 ml buffer 2 (30 mM Tris-HCl, 200 mM KCl, 2 mM DTT, 10% Glycerol, 50 mM imidazole, pH 8.0, with same protease inhibitor), after which the desired protein was finally eluted with 6 ml buffer 3 (30 mM Tris-HCl, 1 M KCl, 2 mM DTT, 10% glycerol, 250 mM imidazole, pH 8.0, plus the protease inhibitor) and collected in 0.5-ml aliquots. Fractions containing the desired protein were pooled, and gel filtration was performed on Superdex 75 or 200 10/300 GL columns (GE Healthcare) mounted into an ÄKTA P100 chromatography system (GE Healthcare). Columns were washed and equilibrated with SE buffer (30 mM Tris-HCl, 200 mM KCl, 2 mM DTT, 10% glycerol, pH 8.0), and 0.5 ml fractions were collected after sample injection. All proteins eluted as a single peak, consistent with their inability to form multimers in the absence of substrate. Protein concentrations were determined using the Bradford assay and protein purity assessed by SDS-PAGE on 12.5% or 15% polyacrylamide gels, followed by Coomassie-Blue staining, with signal from the main band divided by the combined signal from all visible bands, using ImageJ software. The effective concentration of each variant was then inferred from these purity values. Protein concentrations used in subsequent assays were adjusted accordingly. The purified proteins were aliquoted and stored at −80°C. *Drosophila* mtSSB was expressed and purified as described previously (52).

### Nucleic acid substrates

RNA and DNA oligonucleotides used for the experiments are listed in Supplementary Table S1. For testing nuclease activity, DNA or RNA oligonucleotides were 5′ radiolabelled with [γ-^32^P]-ATP (PerkinElmer, 3,000 Ci/mmol), using T4 Polynucleotide Kinase (New England Biolabs) as described in manufacturer’s protocol. The radiolabelled nucleic acid was recovered by gel-filtration using a Sephadex G-50 fine Quick Spin column (Roche) according to manufacturer’s instructions, and its concentration was estimated by scintillation counting. To generate a double-stranded substrate, a 2-fold excess of the complementary strand was added and incubated for 5 min in annealing buffer (90 mM Tris-HCl, 10 mM MgCl_2_, 50 mM KCl, pH 8.0) at 95°C, then bench-cooled to room temperature.

### Nuclease activity assay

Radiolabelled substrate was incubated with purified *Dm* RNase H1, variants or control enzymes as described below and in figure legends, and at the indicated temperatures, in reaction buffer (50 mM Tris-HCl, 2 mM DTT, 5 mM MgCl_2_, 400 μg/ml bovine serum albumin (BSA), pH 8.0) with salt concentration adjusted to 25 mM KCl. Positive control enzymes (ThermoFisher Scientific) were selected according to the substrate: for linear RNA/DNA hybrids and R-loops, RNase H; for dsRNA and ssRNA, RNase A; and for dsDNA and ssDNA, DNase I. Reactions were stopped with 10× STOP solution (10% SDS (w/v), 100 mM EDTA), incubated for 10 min at 60°C, separated by non-denaturing polyacrylamide gel [1× TBE (Tris-borate-EDTA buffer), 12.5% acrylamide:bis-acrylamide 19:1] electrophoresis and heat/vacuum dried for autoradiography (Amersham Hyperfilm ECL, GE Healthcare). Images were analysed densitometrically using ImageJ. For calculating turnover kinetics, initial cleavage rates (*V*_0_) were calculated for each RNase H1 variant at 0.2 nM, in the presence of different concentrations of radiolabelled hybrid (2.5, 5, 7.5 and 10 nM) at different time points. Product generation was plotted as a function of time and *V*_0_ was calculated by estimating the time required to obtain 10% product. *V*_0_ was plotted against substrate concentration and fitted to the Michaelis–Menten equation. Kinetic constants were estimated by the Lineweaver–Burk equation.

### Electrophoretic mobility shift assay

Binding reactions were conducted in binding buffer (25 mM Tris-HCl, 1 mM DTT, 10 mM EDTA, 20 μg/ml BSA, pH 8.0) and salt was adjusted to a final concentration of 25 mM KCl. Protein and nucleic acid concentrations were as indicated in figure legends. Samples were incubated at room temperature for 30 min and fractionated by 6% polyacrylamide gel (0.5× TBE, 6% acrylamide:bis-acrylamide 29:1, 2.5% glycerol) electrophoresis in TBE buffer. Non-radiolabelled nucleic acid was stained with GelRED^TM^ (Biotium) for 20 min in TBE and washed for 20 min with TBE. Gel images were analysed with ImageJ.

### Biolayer interferometry

5′-Biotinylated (RNA or DNA) oligonucleotides (Sigma, Supplementary Table S1) were diluted with non-biotinylated complementary oligonucleotides in PBS, each at a concentration of 10 µM. The oligonucleotide mixture (dsDNA, dsRNA or RNA/DNA hybrid) was incubated at 95°C for 5 min and left to anneal at room temperature overnight. Streptavidin-coated biosensors (FortéBio) were humidified for 30 min in water. This and all subsequent steps were conducted in 384-well plates using 80 μl of solution per well. Sensors were transferred to 25 nM annealed, biotinylated nucleic acid solution for 5 min, followed by a quenching step with 10 µg/ml Biocytin (Sigma) diluted in PBS. Sensors were blocked and equilibrated with Kinetics Buffer (30 mM Tris-HCl, 100 mM KCl, 10% glycerol, 10 mM EDTA, 2 mM DTT, 400 µg/ml BSA, pH 8.0) twice for 10 min. Sensors were transferred to Kinetics Buffer containing different protein concentrations for 15 min. Dissociation was measured using an Octet^®^ RED384 biolayer interferometry (BLI) detection system (FortéBio), by transferring the sensors to Kinetics Buffer for 15 min. All steps were conducted at 30°C with mixing at 1,000 rpm. Processing and analysis of the data were as described (53), using Octet^®^ Systems software (FortéBio). Monovalent (1:1) or heterogeneous (2:1) binding models were used for estimating equilibrium dissociation constant (*K*_D_), association rate (*K*_on_) and dissociation rate (*K*_off_), as indicated in figures and tables for each variant.

### Immunocytochemistry

S2 cell transfection, fixation, staining and imaging were as described previously (2). mtYFP and mtSSB-HA were detected using mouse monoclonal antibodies, respectively, against GFP (Abcam ab1218, 1:10,000), and HA tag (2-2.2.14; ThermoFisher Scientific 26183, 1:10,000), used with goat anti-mouse IgG AlexaFluor^®^568 (ThermoFisher Scientific A-11004, 1:10,000) as secondary antibody. Mitochondrial Cox4 was counter-stained with rabbit polyclonal anti-COXIV antibody (Abcam ab16056, 1:10,000), used with goat anti-rabbit IgG AlexaFluor^®^488 (ThermoFisher Scientific A-11008, 1:10,000) as secondary antibody. Images were optimized for brightness and contrast using ImageJ but not manipulated in any other way.

### Immunoprecipitation from S2 cells

Stably transformed S2 cell-derived cell lines were generated (2) and maintained (51), and protein expression induced as described previously (2). Immunoprecipitation was conducted essentially as described previously (54), using five independent biological replicates from each variant. Approximately, 6 × 10^8^ cells were cross-linked with 1% formaldehyde for 10 min at room temperature with continuous agitation. Cross-linking was stopped by adding 125 mM glycine. Cells were harvested by centrifugation, washed four times with ice-cold Tris-buffered saline, resuspended in RIPA lysis buffer (50 mM Tris-HCl, 150 mM NaCl, 1% v/v Nonidet P40; 0.5% w/v sodium deoxycholate; 0.1% w/v SDS, pH 7.6) and incubated for 30 min on ice. Cells were water-bath sonicated (FinnSonic M0, ultrasonic power 200 W, ultrasonic frequency 40 kHz) at 4°C for 20 min. Cell extracts were incubated at 37°C for 30 min following the addition of RNase A (ThermoFisher Scientific, 100 μg/ml), DNase I (ThermoFisher Scientific, 5 U/ml), Benzonase^®^ nuclease (Sigma, 50 U/ml), MgCl_2_ to 2.5 mM and CaCl_2_ to 1 mM. Samples were centrifuged for 10 min at 4°C, after which supernatants were incubated with 3 μl anti-V5 (Invitrogen R-960-25) or anti-HA (Invitrogen 26183) antibody overnight at 4°C on a rocking shaker. Protein–antibody complexes were collected by incubating protein extracts with 20 μl SureBeads Protein G magnetic beads (Bio-Rad) for 30 min at room temperature on a rocking shaker, followed by three washes with RIPA buffer and resuspension of magnetic beads in protein-loading buffer (PLB: 2% w/v SDS; 2 mM β-mercaptoethanol, 4% v/v glycerol, 40 mM Tris-HCl, 0.01% w/v bromophenol blue, pH 6.8). Cross-linking was reversed by heating at 95°C for 30 min. Samples were analysed by mass-spectrometry, as below, or by Western blotting, essentially as described previously (2), using the following primary/secondary antibodies: rabbit anti-6x-His Tag (ThermoFisher Scientific PA1-983B, 1:10,000), mouse monoclonal anti-GFP [Abcam ab1218, 1:10,000, used to detect yellow fluorescent protein (YFP)] and mouse monoclonal anti-HA Tag (ThermoFisher Scientific 26183, 1:10,000), used with either IRDve^®^ 680LT anti-mouse (LI-COR, 925-68022, 1:10,000) or IRDve^®^ 680LT anti-rabbit (LI-COR, 925-68023, 1:10,000), as appropriate. Blot images were cropped and rotated for presentation, and optimized for contrast and brightness, but not subjected to other manipulations.

### Liquid chromatography coupled to tandem mass spectrometry

Liquid chromatography coupled to tandem mass spectrometry (LC-MS/MS) was carried out on an EASY-nLC1000 chromatograph connected to a Velos Pro-Orbitrap Elite hybrid mass spectrometer with nano-electrospray ion source (all instruments from ThermoFisher Scientific). The LC-MS/MS samples were separated using a two-column setup, consisting of a 2-cm C18 Pepmap column (#164946, ThermoFisher Scientific), followed by a 15-cm C18 Pepmap analytical column (#164940 ThermoFisher Scientific). Samples were loaded in buffer A and the linear separation gradient consisted of 5% buffer B for 5 min, 35% buffer B for 60 min, 80% buffer B for 5 min and 100% buffer B for 10 min at a flow rate of 0.3 μl/min (buffer A: 0.1% trifluoroacetic acid in 1% acetonitrile; buffer B: 0.1% trifluoroacetic acid in 98% acetonitrile). Six microlitres of sample was injected per LC-MS/MS run and analysed. Full MS scans were acquired with a resolution of 60,000 at 300–1,700 *m*/*z* range in the Orbitrap analyser. The method was set to fragment the 20 most intense precursor ions with CID (energy 35). Data were acquired using LTQ Tune software. Acquired MS2 scans were searched against the *Dm* protein database [(55) Uniprot 2017; with 41,170 entries] using the Sequest search algorithms in Thermo Proteome Discoverer (version 1.4). All data were reported based on 95% of confidence for protein identification, *i.e.* a false discovery rate of 5% was applied. Allowed mass error for the precursor ions was 15 ppm, and for the fragment 0.8 Da. A static residue modification parameter was set for carbamidomethyl +57,021 Da (C) of cysteine residue. Methionine oxidation was set as dynamic modification +15,995 Da (M). Only full tryptic peptides were allowed, with a maximum of one missed cleavage.

## Results

### Structure modelling of *Dm* RNase H1

*Dm* RNase H1 was previously proposed to comprise three structural elements (47): an N-terminal HBD domain similar to a region of caulimovirus ORF VI protein (56), an RNase H catalytic domain located towards the C-terminus and a 115-amino acid extended linker that connects these domains. Although the extended linker is longer, the overall architecture is similar to that of RNase H1 from other eukaryotes (Fig. 1A) (20). Structure modelling of the *Drosophila* sequence revealed conserved and potentially novel features of the enzyme. Two of the four amino acids of the human HBD required for hybrid binding (22) are conserved, namely Y29 and K60, contacting the DNA backbone, equivalent to Y19 and K50 in the fly enzyme (Supplementary Fig. S1A). The main-chain amides of human R52 and A55 form hydrogen bonds with 2′-OH groups from two consecutive ribonucleotides. These amino acids are replaced in *Drosophila* by G42 and N45. The DEDD motif at the catalytic core (33) is also present in *Drosophila* at positions D187, E228, D252 and D318 (Supplementary Fig. S1D and F) as are two of the three amino acids of the catalytic domain involved in hybrid binding (27) in human, *i.e.* N151 and N182 (N193 and N224 in *Drosophila*), while the third, human Q183 is represented in *Drosophila* as N225. Protein structure modelling software predicted that a portion of the extended linker could fold as an additional HBD (amino acids 87–142, Supplementary Fig. S1B and C). The conserved N-terminal HBD (amino acids 17–64) shows 42% sequence identity with human RNase H1, with a QMEAN (estimated value of the geometric properties of the model) of 0.55 and a GMQE (being a quality estimation that merges properties of the sequence-template alignment and the template search method) of 0.18. The second predicted HBD has only 24% sequence identity, a QMEAN of −3.32 and GMQE of 0.08 (*48*–*50*). The conserved HBD shows a similar structure as in human (22), with two α-helices and three antiparallel β-sheets, organized as a ββαβα structure. The second predicted HBD is similar, but with longer loops connecting β_1_ to β_2_ and α_1_ to β_3_. The presence of an additional HBD-like domain has previously been reported in *Saccharomyces cerevisiae* (35, 57), where it stabilizes interaction with dsRNA. These observations prompted us to explore the binding properties of the different domains of the *Drosophila* enzyme.

### Enzymatic properties of *Drosophila* RNase H1

To explore its biochemical properties, the full-length *Dm* RNase H1 protein was expressed in *E. coli*. The expressed protein consisted of 316 amino acids from the RNase H1 open-reading frame, commencing at A17, assuming post-translational removal of the N-terminal formyl-methionine by the action of methionine aminopeptidase (58), plus an additional eight amino acids at the C-terminus from the 6x-His tag and two further amino acids contributed by the vector, pET-26b(+). This expressed protein represents the nuclearly targeted variant, which is very similar to the mitochondrially targeted variant after removal of the predicted mitochondrial targeting signal. Following affinity purification and size-exclusion chromatography, it migrated on SDS-PAGE gels with an apparent molecular mass of ∼38 kDa (Fig. 1C), close to prediction (35.1 kDa plus the C-terminal tag). We initially investigated the kinetic properties of the enzyme at 30°C, using a blunt-ended model substrate comprising a 5´-radiolabelled 30 nt RNA oligonucleotide hybridized to a complementary 30 nt DNA oligonucleotide (Table I; for original gels and graphical plots see Supplementary Fig. S2A–C). For comparison with the previously studied human enzyme, we reinvestigated RNase H1 from the two species at both 30 and 37°C. At 30°C, the human enzyme had no detectable activity, while at 37°C it performed as previously reported (34), validating the methods employed. The *Drosophila* enzyme showed a greatly increased *K*_M_ but also an approximate doubling of *K*_cat_ at 37°C, compared with its properties at 30°C. Note that 37°C is far above the physiological temperature range for the fly enzyme *in vivo* (15–30°C). In comparison with the human enzyme at 37°C, the *Drosophila* enzyme at 30°C can be regarded as considerably more active, with a much lower *K*_M_ and much higher *K*_cat_ (Table I and Supplementary Fig. S2). The prediction of a second HBD and the high enzymatic activity of the enzyme *in vitro* prompted us to look next at its ability to bind RNA/DNA hybrid.

**Table:** Table I. **Estimated kinetic parameters^a^ of RNase H1 and variants**

Enzyme	*K*_M_ (nM)	*K*_cat_ (min^−1^)
Human RNase H1 (37°C)	42 ± 8.2	3.0 ± 1.5
*Dm* RNase H1 (37°C)	170 ± 5.7	27 ± 2.5
*Dm* RNase H1 (30°C)	8.5 ± 1.8	13 ± 3.3
ΔI/II (30°C)	4.0 ± 1.1	13 ± 7.7
ΔII/III (30°C)	3.1 ± 0.55	1.6 ± 1.6
ΔI–III (30°C)	9.7 ± 1.8	30 ± 9.0
*Dm* RNase H1 D252N (30°C)	0	0

a Means ± SD (*n* ≥ 3) for *Dm* and human enzymes and variants. All values are given to two significant figures.

### Hybrid-binding properties of *Dm* RNase H1

**Fig. 2.**
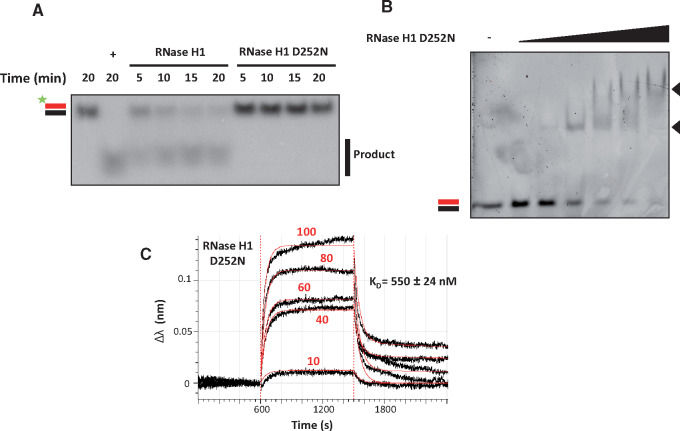
**Enzymatic analysis of *Dm* RNase H1.** (**A**) In a 10 μl reaction, 100 fmol of 30-bp 5′-radiolabelled RNA/DNA hybrid were incubated with 2 fmol of RNase H1 or RNase H1 D252N (catalytically inactive) for the indicated times (min), and separated by non-denaturing gel electrophoresis. In this and subsequent figures RNA/DNA hybrid is denoted by parallel red (online edition only) (RNA) and black (DNA) bars, with radiolabel indicated by the green asterisk (online edition only). Positive control (+) used *E. coli* RNase H. (**B**) In a 20 µl reaction, 2 pmol of a 30-bp RNA/DNA hybrid were incubated with increasing concentrations of RNase H1 D252N (0, 0.1, 0.5, 1, 2.5, 5 and 10 µM) and separated by non-denaturating gel electrophoresis. Black arrowheads indicate two different protein–nucleic acid complexes. (**C**) BLI analysis of catalytically inactive (D252N) RNase H1 interacting with linear 30-bp RNA/DNA hybrid. Streptavidin sensors were incubated in 80 µl of 25 nM 5′ biotinylated hybrid solution. Association was measured by transferring sensor to 80 µl of different concentrations of RNase H1 D252N solution [0, 10, 40, 60, 80 and 100 nM, as indicated in red text (online edition only)]. Values for the reference sample (0 nM) were subtracted before plotting. The sensogram displays the baseline, association and dissociation steps, with experimental data shown as black lines and extrapolated data fitted to heterogeneous (2:1) binding model as red lines (online edition only). The inferred *K*_D_ for the first binding step is shown (see Table II for estimated association/dissociation parameters).

To study the binding of *Dm* RNase H1 and its derivatives to nucleic acid substrates, we generated a single point mutation (D252N) at the catalytic core, which abolished enzymatic activity (Fig. 2A). This enabled us to use an electrophoretic mobility shift assay (EMSA) to profile qualitatively its nucleic acid-binding properties. A 10-fold molar excess of the protein was sufficient to generate a detectable complex with a 30-bp RNA/DNA hybrid, with a secondary mobility shift seen at higher protein/hybrid ratios (Fig. 2B). Trials with substrates of different lengths down to 22 bp also revealed a second mobility shift at high protein concentrations (Supplementary Fig. S2D), suggesting the formation of higher-order complexes, most likely two protein moieties with one substrate. Substrate-binding kinetics were then analysed quantitatively in real time by BLI, the degree of resolution by EMSA being insufficient to extrapolate kinetic parameters (53).

The RNA/DNA hybrid substrate was immobilized on the sensors and the binding of the enzyme at various concentrations was analysed. The shapes of the association and dissociation curves (Fig. 2C) suggest heterogeneous binding, which was also inferred by the instrument software, fitting the data preferentially to a 2:1 binding model, *i.e.* with the enzyme binding in two steps to the substrate. The estimated affinity of the first binding step was relatively low, with *K*_D_ = 550 ± 24 nM, but much stronger in the second, with *K*_D_ = 0.23 ± 0.063 nM (Table II). These data suggest that the first binding step represents substrate recognition, whereas the second step likely stabilizes the enzyme on the substrate. For comparison, the *K*_D_ for the second binding step is ∼10-fold lower than that reported for mtSSB from *Dm* in its binding to ssDNA (2.5 nM) (59). To dissect these findings in more detail and relate them to the structure of the enzyme, we next analysed the enzymatic and hybrid-binding properties of *Dm* RNase H1 variants bearing deletions of specific domains.

**Table:** Table II. **Estimated kinetic parameters^a^ of binding of RNase H1 variants to different substrates**

Nucleic acid	Enzyme	*K*_D1_ (nM)	*K*_D2_ (nM)	*K*_on1_ (1/M*s)*10^−3^	*K*_on2_ (1/M*s)*10^−3^	*K*_off1_ (1/s)*10^3^	*K*_off2_ (1/s)*10^3^
Linear hybrid	RNase H1	550 ± 24	0.22 ± 0.063	33 ± 1.4	490 ± 9.1	18 ± 0.061	0.11 ± 0.0023
	ΔII/III	110 ± 0.75	0.65 ± 0.018	220 ± 1.4	170 ± 0.99	24 ± 0.044	0.11 ± 0.0029
dsRNA	RNase H1	2,200 ± 170	750 ± 6.2	20 ± 15	0.40 ± 0.0022	440 ± 12	0.30 ± 0.0019
	ΔII/III	2,100 ± 30	940 ± 160	150 ± 21	0.66 ± 0.11	310 ± 1.9	0.62 ± 0.030
dsDNA	RNase H1	13,000 ± 910	340 ± 6.6	36 ± 0.23	1.8 ± 0.026	480 ± 8.4	0.62 ± 0.075
	ΔII/III	1,900 ± 54	120 ± 6.3	190 ± 5.0	0.63 ± 0.0070	370 ± 3.5	0.073 ± 0.0039

a Based on BLI, using a heterogeneous (2:1) binding model for these variants, which gave the best fit. All values are given to two significant figures.

### The HBD domains influence *Dm* RNase H1 activity

**Fig. 3.**
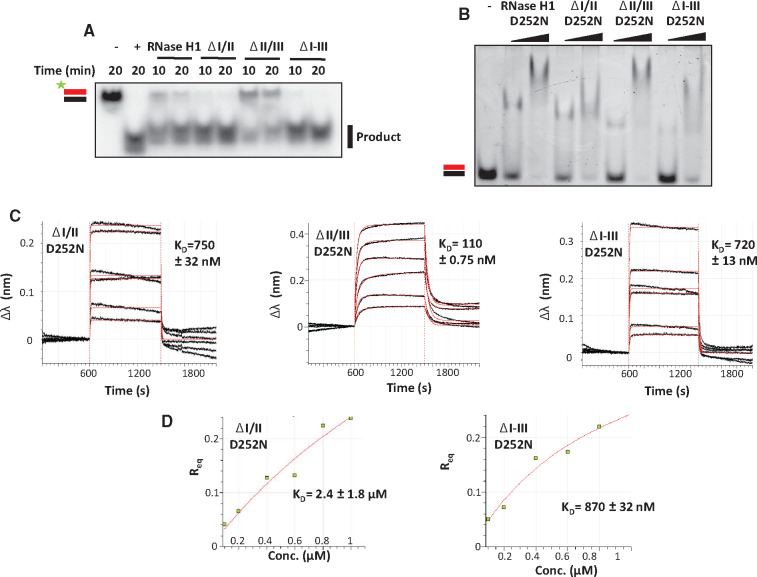
**Biochemical analysis of *Dm* RNase H1 variants**. (**A**) Enzymatic assay, (**B**) EMSA and (**C**, **D**) BLI, using *Dm* RNase H1 variants as indicated. Other details as for Fig. 2, except protein concentrations in (B) were 1 and 10 μM. Positive control in (A) used *E. coli* RNase H (+). For BLI analysis, sensors were incubated in increasing concentrations of RNase H1 variant proteins (ΔI/II and ΔI–III: 0, 100, 200, 400, 600, 800 and 1,000 nM; ΔII/III: 0, 10, 20, 40, 60, 80 and 100 nM). Values for the reference sample (0 nM) were subtracted before plotting. The red lines (online edition only) of the sensograms shown in (C) are extrapolated data fitted to a monovalent (1:1) binding model (left and right sensograms, for ΔI/II and ΔI–III variants, respectively) or to a heterogeneous (2:1) binding model (middle sensogram, for ΔII/III variant). The inferred *K*_D_ for the first (or only) binding step in each case is shown (see Tables II and III for association/dissociation parameters). (D) Steady-state analysis of relative equilibrium (*R*_eq_) plotted against protein concentration, for the variants that fitted a 1:1 binding model. Although less precise, this method of analysis produced similar estimated dissociation constants ± standard deviation (SD), as shown.

For the purposes of this analysis we defined five regions of the enzyme, from N- to C-terminus (Fig. 1B), as follows: Region I is the conserved HBD (amino acids 17–64), Region II (amino acids 65–86) is the short linker leading up to the second predicted HBD, Region III (amino acids 87–141). Region IV is another short linker (amino acids 142–178), leading up to Region V, the RNase H catalytic domain (amino acids 179–333). Variants lacking Region IV but retaining Region V, as well as the one comprising only Region III, were insoluble, even when expressed using an auto-induction system and tested under different buffer conditions (salt, pH), and thus were not studied further. All purified variant proteins migrated on SDS-PAGE gels approximately as predicted (Fig. 1C). The measured kinetic parameters of those variants that retained detectable catalytic activity are indicated in Table I (Supplementary Fig. S2). In summary, deletion of Regions I–III (ΔI–III) or of just the conserved HBD and its adjacent linker (ΔI/II) facilitated catalysis (Fig. 3A), though with subtly different effects on the kinetic parameters (Table I and Supplementary Fig. S2), whereas deletion of just the second predicted HBD and its upstream linker (ΔII/III) caused almost a 90% decrease in *K*_cat_ at 30°C, despite a decreased *K*_M_ (Table I, Fig. 3A and Supplementary Fig. S2). In more general terms, the conserved HBD, whenever it was present, appeared to restrain catalysis. However, the second HBD mitigated this effect. Both the I/II and II/III regions decreased substrate binding to the catalytic centre, as inferred from lower *K*_M_ values in their absence. This might be a simple consequence of the presence of additional binding steps preceding the loading of the substrate to the catalytic centre. In addition, the II/III region appears to support an efficient turnover rate, as its absence results in a decrease of the *K*_cat_ value by ∼8-fold (Table I). A simultaneous decrease in both *K*_M_ and *K*_cat_, as observed in the absence of the II/III region (Table I), is indicative of a decreased rate of dissociation of the enzyme-product complex. In turn, this implies that the second HBD may be relevant for effective product release. Given that the RNase has to progress along the substrate, the binding properties of  Region III might be relevant to the translocation process. The lack of both HBD domains resulted in a >2-fold increase in turnover rate (Table I), which implies that substrate binding by the HBD domains together limits the rate of catalysis. To explore this further, we then looked at the hybrid-binding properties of the variants lacking either or both HBDs.

### Hybrid-binding properties conferred by the HBDs

We proceeded to test the hybrid-binding properties of these variants by introducing the D252N mutation. Analyses by EMSA (Fig. 3B) and BLI (Fig. 3C and Table II) showed that the ability to bind RNA/DNA hybrid was retained when the conserved (ΔI/II D252N) or second HBD (ΔII/III D252N) were deleted, or even when both HBDs were deleted (ΔI–III D252N). However, the kinetic parameters of the binding reactions, as inferred by BLI, indicated a difference in the nature of the binding to the two HBDs. Wherever the conserved HBD was missing, the sensogram indicated a 1:1 substrate-binding model (Fig. 3 and Table III), rather than the 2:1 model (Figs 2 and 3 and Table II) that was more compliant with the data from full-length RNase H1 or the variant lacking only the second HBD (ΔII/III D252N). The ‘supershift’ band observed by EMSA with the full-length protein at high protein:substrate ratio (Fig. 2B and Supplementary Fig. S2D) was also abolished when the conserved HBD was absent (Fig. 3B).

**Table:** Table III. **Estimated kinetic parameters^a^ of binding to 30-bp linear hybrid^b^, of RNase H1 variants lacking the conserved HBD or catalytic domain**

Enzyme variant	*K*_D_ (nM)	*K*_on_ (1/M*s)*10^−3^	*K*_off_ (1/s)*10^3^
ΔI/II	750 ± 32	200 ± 8.0	150 ± 2.0
ΔI–III	720 ± 13	110 ± 1.8	79 ± 0.44
ΔII–V	140 ± 0.81	110 ± 0.64	15 ± 0.029
ΔIII–V	170 ± 0.91	33 ± 0.16	5.6 ± 0.0093
ΔIV/V	370 ± 3.0	62 ± 0.43	23 ± 0.055

a Based on BLI, using a (1:1) binding model for these variants, which gave the best fit. All values are given to two significant figures.

b These variants all showed no detectable binding to dsRNA or dsDNA (see Supplementary Figs S3, S4 and S6).

The deletion of region I/II (conserved HBD) resulted in an ∼35% decrease in substrate-binding affinity (compare Fig. 3C and D, Table III with Fig. 2C, Table II), consistent with the role of this domain in substrate binding inferred from the lower *K*_M_. Conversely, the lack of region II/III (the second HBD) substantially increased substrate affinity (∼5-fold). Taken together with the decreased *K*_cat_, this strengthens the case that the second HBD is needed for efficient substrate release and thus, most likely, for the enzyme’s translocation along the substrate. The full N-terminal region (I–III) truncation behaved in a similar manner to the variant lacking only the conserved HBD (Table III), implying that the second HBD only functions in co-operation with the first. As RNase H1 must interact with a template composed mostly of dsDNA, and which is also actively transcribed, we next investigated whether the *Drosophila* enzyme was able to bind to other nucleic acid substrates than RNA/DNA hybrid.

### Binding of *Dm* RNase H1 to other nucleic acid substrates

**Fig. 4.**
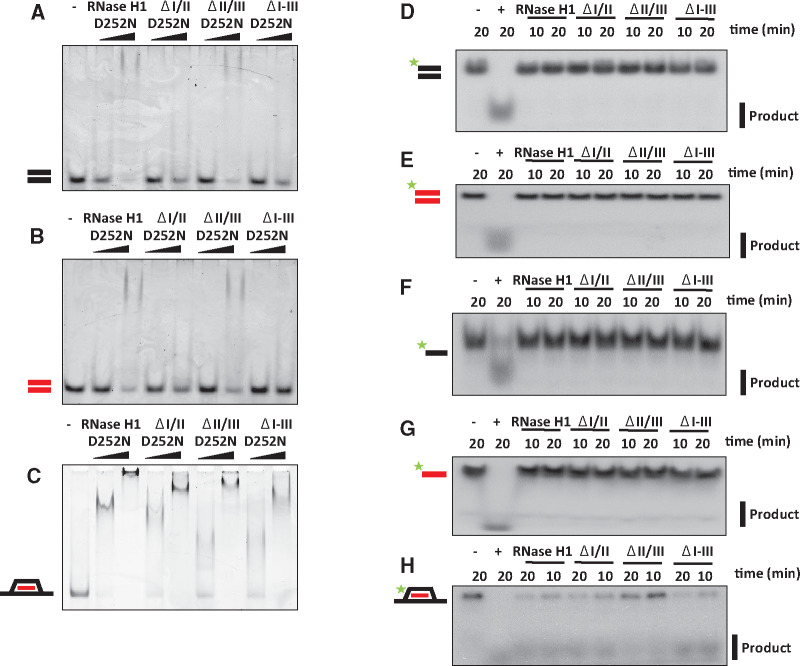
***Dm* RNase H1 binds but does not degrade non-hybrid double-stranded nucleic acid**. (**A**–**C**) EMSA and (**D**–**H**) nuclease assays using *Dm* RNase H1 variants and substrates as indicated, according to the nomenclature of Fig. 2. Protein concentrations in (A–C) as for Fig. 3, other details as for Fig. 2. Positive control (+) in (D, F) DNase I, (E, G) RNase A and (H) *E. coli* RNase H.

We next considered the broader nucleic acid-binding properties and substrate preferences of the enzyme. EMSA analysis using the D252N-substituted variants revealed that the full-length protein as well as deletion constructs lacking the second HBD (ΔII/III) were able to bind both dsDNA (Fig. 4A) and dsRNA (Fig. 4B), although this binding was weakened substantially when the conserved HBD (ΔI/II) or both HBDs (ΔI–III) were deleted (Fig. 4A and B). An R-loop substrate was bound by all of these constructs, including the catalytic domain alone, together with the preceding short linker (ΔI–III; Fig. 4C), and in each case a supershift was observed at high protein concentration (Fig. 4C). BLI (Table II) confirmed these findings, although the deletion of the second HBD increased the binding affinity for dsDNA, but not dsRNA (Supplementary Figs S3 and S4). The affinity of all of the tested variants for RNA/DNA hybrid was at least 1–2 orders of magnitude greater than for dsRNA or dsDNA (Tables II and III). Neither the full-length D252N-substituted protein, nor any of the variants tested, had any detectable affinity for ssRNA or ssDNA (Supplementary Fig. S5) nor did the equivalent variants without the D252N substitution show any detectable nuclease activity against dsDNA (Fig. 4D), dsRNA (Fig. 4E), ssDNA (Fig. 4F) or ssRNA (Fig. 4G). Removal of the second HBD appeared to have a negative effect on catalytic activity using the R-loop substrate (Fig. 4H), although removal of the conserved HBD had no major effect on this activity, which differed from the effect seen using the linear RNA/DNA substrate (Fig. 3A). Another difference between the linear hybrid and R-loop substrates was that the constructs lacking the conserved HBD still produced an EMSA supershift using the latter substrate (Fig. 4C). The binding studies described above indicated a role for the catalytic domain in both hybrid and dsDNA binding. To investigate this further, we created a deletion series in which the catalytic domain was removed.

### Effects on nucleic acid-binding of deleting the catalytic domain

**Fig. 5.**
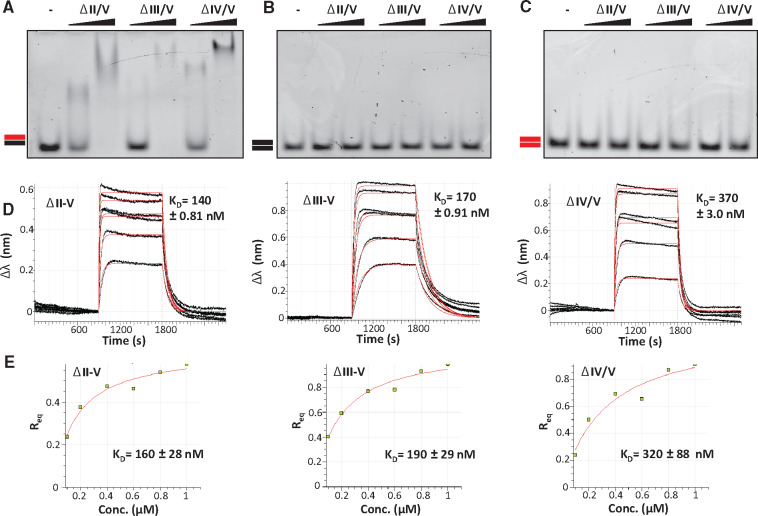
**Catalytic domain influences nucleic acid-binding properties of *Dm* RNase H1**. (**A**–**C**) EMSA and (**D**, **E**) BLI using *Dm* RNase H1 variants and substrates as indicated, according to the nomenclature of Fig. 2 and 3. The red lines (online edition only) of the sensograms shown in (D) are extrapolated data fitted to a monovalent (1:1) binding model (see Table III for association/dissociation parameters). (E) Steady-state analysis of relative equilibrium (*R*_eq_) plotted against protein concentration. Protein concentrations in (A–C) as for Fig. 3, other details as for Fig. 2.

We studied the binding properties of D252N-substituted variants ΔII–V, ΔIII–V and ΔIV/V, lacking the catalytic domain. In EMSA, all of these variants bound RNA/DNA hybrid and showed a supershift at high protein concentration (Fig. 5A). However, none of them bound dsDNA (Fig. 5B and Supplementary Fig. S6A) or dsRNA (Fig. 5C and Supplementary Fig. S6B). Quantitative analysis by BLI (Fig. 5D and E and Table III) showed that these variants all bound hybrid more tightly than the full-length protein, with the conserved HBD alone (ΔII–V) giving the strongest binding. Overall, these findings confirm that the conserved HBD confers tight binding to hybrid, while the combination of the conserved HBD and the catalytic domain (plus its immediately upstream linker) is needed for the much weaker binding to dsDNA or dsRNA. In contrast, the second HBD weakens binding both to hybrid and to dsDNA (Table II), consistent with its proposed role in promoting dissociation from the product and facilitating processivity.

### Protein interactors with *Dm* RNase H1

The properties of the different regions of *Dm* RNase H1 are strikingly distinct, perhaps reflecting the fact that the enzyme must operate in two different cellular compartments and is implicated in a variety of macromolecular processes (see Introduction). To gain further insight into the physiological roles of the protein and its various domains, we initiated a shotgun proteomic screen for proteins that bind to the enzyme. Aiming to identify specific molecular interactors, we applied a whole-cell cross-linking approach (54), initially with just one round of immunopurification, using as bait the full-length protein. As a control to exclude proteins appearing in the list due to non-specific proteotoxic stress provoked by overexpression of a protein targeted to mitochondria, we included mitochondrially targeted YFP (Supplementary Fig. S7). Based on the results of the previous study in which we profiled the dual targeting of the enzyme in S2 cells (2), we included also the two variants showing the most compartment-specific localization, M1V (nuclear) and M16V (predominantly mitochondrial). Finally, with the intent of trapping proteins interacting only transiently with *Dm* RNase H1 during the catalytic cycle, we included also the D252N variant. Raw data for each replicate (submitted to MassIVE data repository, accession number MSV000085303) are shown in Supplementary Table S2. In each case, we retained target proteins that were identified in every replicate experiment with the given bait protein (*n* = 5 in each case), but which and were absent from all controls (60). The primary screen revealed a list of 63 proteins that we subdivided into two main groups, nuclear (Table IV) and mitochondrial candidates (Table V), based on the major subcellular location of the protein as currently annotated in Flybase (www.flybase.org). In general, nuclear candidates were found using the full-length, D252N and M1V variants, whereas the mitochondrial candidates were negative using M1V, but few were detected by M16V either, possibly an issue with expression level. The nuclear candidate list included proteins with previously known or inferred roles in heteroduplex processing and DNA replication, whereas the mitochondrial candidates covered a wider spectrum, including many metabolic enzymes not previously implicated in nucleic acid metabolism. Both lists included the respective, compartment-specific single-stranded DNA-binding proteins, RpA-70 (as previously reported in mammals) (24) and mtSSB. Recently, based on *in vitro* studies of the mammalian proteins, it was proposed that mtSSB and RNase H1 collaborate to generate an RNA primer that would be used by Polγ to initiate mtDNA replication (10). This, and the paucity of other proteins with known or hypothesized roles in DNA transactions among the mitochondrial candidates, led us to investigate the interaction between mtSSB and *Dm* RNase H1 in more detail.

**Table:** Table IV. **List of nuclear candidates for *Dm* RNase H1 interactors**

Category	CG number^a^	Official name, gene symbol^a^	Human orthologue^a^	Mean PSM factor	Detected by which variant(s)?			
					RNase H1	D252N	M1V	M16V
Genome maintenance and transcription	CG10279	Rm62, isoform H, *Rm62*	DDX5, DDX17	9	Yes	Yes	Yes	No
	CG9633	Replication protein A 70, *RpA-70*	RPA1	6.6	No	No	Yes	No
	CG7831	Non-claret disjunctional, *ncd*	KIFC1	4	No	No	Yes	No
	CG5499	Histone H2A variant, *His2Av*	H2AFV, H2AFZ	3	No	No	Yes	No
	CG4747	Nucleosome-destabilizing factor, *Ndf*	GLYR1	2.6	No	No	Yes	No
	CG4206	Minichromosome maintenance 3, *Mcm3*	MCM3	1.8	No	No	Yes	No
	CG7538	Minichromosome maintenance 2, *Mcm2*	MCM2	1	No	No	Yes	No
Cell cycle progression	CG6392	CENP-meta, *cmet*	CENPE	1	Yes	No	No	No
Other	CG5436	Heat shock protein 68, *Hsp68*	HSPA1A/HSPA1B	4.8	Yes	No	Yes	Yes

a Based on current information in Flybase (flybase.org).

**Table V.** List of mitochondrial candidates for *Dm* RNase H1 interactors

Category	CG number^a^	Official name, gene symbol^a^	Human orthologue^a^	Mean PSM factor	Detected by which variant(s)?			
					RNase H1	D252N	M1V	M16V
Metabolism	CG10622	Succinyl-coenzyme A synthetase β subunit, GDP-forming, *ScsβG*	SUCLG2	9.6	Yes	Yes	No	Yes
	CG7010	Pyruvate dehydrogenase E1 component subunit alpha, *Pdha*	PDHA2	7.6	Yes	Yes	No	Yes
	CG8778	CG8778 [enoyl-CoA hydratase], *CG8778*	AUH^b^	6.2	Yes	Yes	No	No
	CG7920	CG7920, isoform A, *CG7920*	[MATN2]^c^	6	Yes	Yes	No	Yes
	CG6439	Isocitrate dehydrogenase 3b, *Idh3b*	IDH3B	6	Yes	No	No	No
	CG12262	Medium-chain acyl-CoA dehydrogenase, *Mcad*	ACADM	5	Yes	No	No	No
	CG4703	Arc42 [Short-chain acyl-CoA dehydrogenase], *Arc42*	ACADS	4.8	Yes	Yes	No	Yes
	CG5889	Malic enzyme, *Men-b*	ME3	4.8	Yes	No	No	No
	CG9006	Enigma, *Egm*	ACAD9	3.8	Yes	Yes	No	No
	CG5320	Glutamate dehydrogenase, *Gdh*	GLUD1	3.6	Yes	No	No	No
	CG5599	CG5599, *CG5599*	DBT^d^	3.4	Yes	No	No	No
	CG5590	CG5590, *CG5590*	HSDL2	3	Yes	Yes	No	No
	CG10639	L-2-hydroxyglutarate dehydrogenase, *L2HGDH*	L2HGDH	3	Yes	No	No	No
	CG16935	CG16935 [Trans-2-enoyl-CoA reductase (NADPH)], *CG16935*	MECR	2.6	Yes	Yes	No	No
	CG1236	CG1236 [Glyoxylate and hydroxypyruvate reductase], *CG1236*	GRHPR	2.6	Yes	No	No	No
	CG4860	CG4860 [Short-chain acyl-CoA dehydrogenase], *CG4860*	ACADS	2.2	Yes	No	No	No
	CG10194	CG10194, *CG10194*	NUDT19	2.2	Yes	No	No	No
	CG5028	CG5028, [Isocitrate dehydrogenase (NAD(+))], isoform C, *CG5028*	IDH3G	2	Yes	Yes	No	No
	CG7842	bad egg, *beg*	MCAT	2	Yes	No	No	No
	CG12140	Electron transfer flavoprotein-ubiquinone oxidoreductase, *Etf-QO*	ETFDH	1.8	Yes	No	No	No
	CG4094	Fumarase 1, *Fum1*	FH	1.8	Yes	No	No	No
	CG10672	CG10672 [Carbonyl reductase (NADPH)], *CG10672*	DHRS4	1.6	Yes	No	No	No
	CG3267	Methylcrotonoyl-CoA carboxylase 2, *Mccc2*	MCCC2	1.6	Yes	No	No	No
Translation	CG6050	mitochondrial elongation factor Tu, *mEFTu1*	TUFM	8.2	Yes	Yes	No	Yes
	CG6412	Mitochondrial elongation factor Ts, *mEFTs*	TSFM	2.4	Yes	Yes	No	No
	CG2957	Mitochondrial ribosomal protein S9, *mRpS9*	MRPS9	2.2	Yes	No	No	No
	CG13126	CG13126, *CG13126*	METTL17	2	Yes	No	No	No
	CG5242	Mitochondrial ribosomal protein L40, *mRpL40*	MRPL40	1.6	Yes	No	No	No
	CG5012	Mitochondrial ribosomal proteinL12, *mRpL12*	MRPL12	1.4	No	Yes	No	No
	CG7494	Mitochondrial ribosomal protein L1, *mRpL1*	MRPL1	1.2	Yes	No	No	No
Protein import and processing	CG11779	CG11779, *CG11779*	TIMM44	8.2	Yes	Yes	No	No
	CG8728	CG8728, *CG8728*	PMPCA	6.6	Yes	Yes	No	No
	CG6155	Roe1, *Roe1*	GRPEL1	3	Yes	Yes	No	No
	CG3107	CG3107, *CG3107*	PITRM1	2	Yes	No	No	No
OXPHOS	CG2286	NADH-ubiquinone oxidoreductase 75 kDa subunit, *ND-75*	NDUFS1	7.6	Yes	Yes	No	No
	CG1970	NADH dehydrogenase (ubiquinone) 49 kDa subunit, *ND-49*	NDUFS2	2.2	Yes	Yes	No	No
	CG14724	Cytochrome c oxidase subunit 5A, *COX5A*	COX5A	1.4	Yes	No	No	No
	CG10340	CG10340, *CG10340*	ATPAF1	1	Yes	No	No	No
	CG3214	NADH dehydrogenase (ubiquinone) B17.2 subunit, *ND-B17.2*	NDUFA12	1	Yes	No	No	No
tRNA metabolism	CG7479	Leucyl-tRNA synthetase, mitochondrial, *LeuRS-m*	LARS2	5.4	Yes	No	No	No
	CG16912	Tyrosyl-tRNA synthetase, mitochondrial, *TyrRS-m*	YARS2	1.2	Yes	No	No	No
mtDNA maintenance	CG4337	Mitochondrial single-stranded DNA-binding protein, *mtSSB*	SSBP1	4.2	Yes	Yes	No	No
Oxidative stress	CG5826	Peroxiredoxin 3, *Prx3*	PRDX3	3	Yes	Yes	No	No
	CG7217	Peroxiredoxin 5, *Prx5*	PRDX5	3	Yes	Yes	No	No
	CG10964	Sniffer, *sni*	RDH5	1	Yes	No	No	No
Other	CG8479	Optic atrophy 1 orthologue, isoform D, *Opa1*	OPA1	6.4	Yes	No	No	No
	CG14434	CG14434, *CG14434*		4	Yes	No	No	No
	CG13850	CG13850, *CG13850*	TBRG4	4	Yes	No	No	No
	CG2794	CG2794, *CG2794*		3.4	Yes	No	No	No
	CG11624	Ubiquitin-63E, *Ubi-p63E*	UBC	3	No	Yes	No	No
	CG5844	Spliceosome–ribosome linker protein, *Srlp*		2.6	Yes	Yes	No	No
	CG5915	Rab7, *Rab7*	RAB7A	1.8	Yes	No	No	Yes
	CG11267	CG11267 [Hsp10 chaperonin subunit], *CG11267*	HSPE1	1.4	Yes	Yes	No	No
	CG8993	CG8993, *CG8993*	TXN2	1	Yes	No	No	No

a Based on current information in Flybase (flybase.org). Some of these genes are still officially identified only by CG numbers, although orthology indicates a clear enzymatic function also reported in Flybase, and shown here [in brackets].

b Human orthologue is an RNA-binding variant of the metabolic enzyme.

c Closest match but does not fulfil all criteria for being a true orthologue.

d Human orthologue is dihydrolipoamide-branched chain transacylase E2.

### *Dm* mtSSB does not interact directly and stably with RNase H1 *in vivo*

**Fig. 6.**
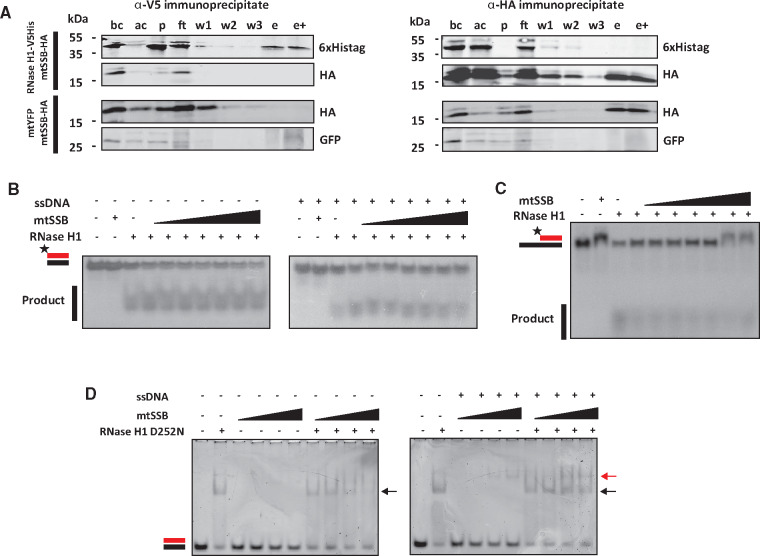
**Mitochondrial single-strand DNA-binding protein does not stimulate RNase H1 activity *in vitro***. (**A**) Western blots of immunoprecipitates from S2 cells co-expressing RNase H1-V5/His and mtSSB-HA or mtSSB-HA and mtYFP. Immunoprecipitates using anti-V5 (left-hand panels) or anti-HA (right-hand panels) from cells transfected as indicated and probed as shown to the right of blot panels. All protein extracts were tracked by successive sampling during the procedure, indicated as follows: bc, before cross-linking; ac, after cross-linking; p, pellet; ft, flowthrough; w1, 2 and 3, washes; e, eluate, still cross-linked; e+, eluate after reversal of cross-linking. Samples were imaged by Western blot using anti-HA for detecting mtSSB-HA, anti-6x-His tag for detecting RNase H1-V5/His and anti-GFP for detecting YFP. (**B**, **C**) Nuclease assays using 0.1 nM of *Dm* RNase H1 and radiolabelled substrates as illustrated, according to the nomenclature of Fig. 2, pre-incubated with increasing concentration of mtSSB (0, 5, 10, 25, 50, 100, 250 and 500 nM), without (B, left panel and C) or with (B, right panel) a 60-nt ssDNA oligonucleotide for 10 min. Densitometric analysis of the gel image shown in (B) indicated a 55% stimulation in activity at the highest dose of SSB used, compared with no SSB (based on the relative intensities of the substrate band and low-molecular-weight product). Substrate in (C) had a 60-nt ssDNA 3′ overhang. (**D**) EMSA reactions with 1 µM RNase H1 D252N variant, incubated with increasing concentrations (1, 2.5, 5, 7.5 μM) of free mtSSB (left panel) or ssDNA + mtSSB complex (right panel), for 10 min at room temperature. Arrowheads indicate the complex formed between RNase H1 and the 30-bp RNA/DNA hybrid (black) and between mtSSB and the 60-nt ssDNA (red, online edition only).

Because mass spectrometry revealed mtSSB as a potential interactor with *Dm* RNase H1, we performed co-immunoprecipitation experiments on S2 cells overexpressing V5/His epitope-tagged RNase H1, HA epitope-tagged mtSSB and mtYFP (as control). The subcellular localization of mtSSB-HA and mtYFP were validated by immunocytochemistry (Supplementary Fig. S7). Western blot analysis revealed that the proteins co-immunoprecipitated with RNase H1-V5/His did not include detectable amounts of mtSSB-HA (Fig. 6A and Supplementary Fig. S8, left-hand panels). Similarly, the proteins co-immunoprecipitated with mtSSB-HA did not include detectable amounts of RNase H1-V5/His (Fig. 6A and Supplementary Fig. S8, right-hand panels).

Despite this negative finding with the epitope-tagged proteins *in vivo*, we investigated the functional interactions of the two purified proteins *in vitro*. Addition of mtSSB in increasing molar excess (50-fold and more) showed a trend towards a modest stimulatory effect (∼50%) on the activity of RNase H1, based on densitometry (see Fig. 6B), although this was abolished when the assay was conducted in the presence of ssDNA (Fig. 6B). When a region of ssDNA was incorporated into the RNase H1 substrate (Fig. 6C), mtSSB had little effect or may even have inhibited the RNase H1 reaction slightly (Fig. 6C), while RNase H1 had no reproducible effect on complex formation between ssDNA and mtSSB (Fig. 6D). A large molar excess of mtSSB also had only a modest effect on the formation of complexes between RNA/DNA hybrid and catalytically inactive RNase H1 (Fig. 6D), regardless of the presence of ssDNA.

## Discussion

**Fig. 7.**
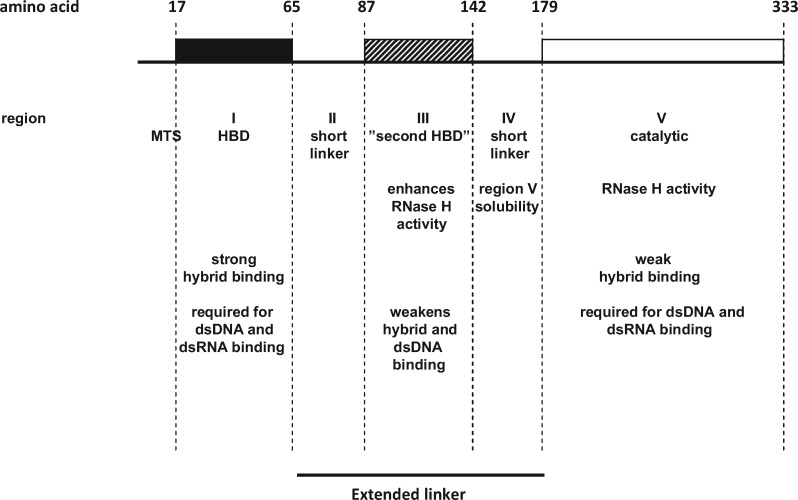
**Functional summary diagram of *Dm* RNase H1**. Schematic representation of *Drosophila melanogaster* RNase H1. The five regions are bounded as shown, by amino acids 17, 65, 87, 142, 179 and 333. The black box represents the conserved HBD, the hatched box the second HBD and the empty box the catalytic domain. Each region has a short description of its function, based on the experimental findings.

In this study, we evaluated the biochemical properties of *Dm* RNase H1, determined the functional roles of each region of the protein and probed for interacting proteins from the two cellular compartments in which RNase H1 is localized. The enzymatic properties of the *Drosophila* enzyme are broadly similar to those of that from humans. However, the two enzymes exhibit different temperature dependencies, but both are highly temperature sensitive. We identified strong binding for RNA/DNA hybrid in the conserved HBD (Region I) and weaker hybrid binding to the catalytic domain (Region V). The HBD also exhibited weak binding for dsDNA and dsRNA, but only in constructs also retaining the catalytic domain. The presence of the intervening domain (III), which we postulated initially as being a second HBD based on structure predictions, had a negative effect on overall hybrid or dsDNA binding affinity, but was also required for full catalytic activity in the presence of the conserved HBD. These properties are summarized in Fig. 7. We identified one major interacting protein from mitochondria, mtSSB. However, studies *in vivo* (Fig. 6A and Supplementary Fig. S8) and *in vitro* (Fig. 6B–D) suggest that the interaction is either transient or indirect, requiring the involvement of other proteins or nucleic acid moieties to mediate or stabilize it.

### Functional characterization of the domains of *Dm* RNase H1

Aiming to understand the role of each domain of the enzyme, we created a series of deletion constructs, having divided *Dm* RNase H1 into five regions, in order to study the properties conferred by each of them on the enzyme (Fig. 1). Despite the absence of conserved amino acids proposed to be involved in ribonucleotide binding, Region I, the conserved HBD, bound RNA/DNA hybrid with a similar affinity as the human HBD (22). The conserved HBD was also required for dsRNA and dsDNA binding (Fig. 4) but, in contrast to the human HBD, it requires the additional presence of the catalytic domain (Regions IV and/or V) for these substrates to be bound (Fig. 5 and Supplementary Fig. S6). The changes in enzyme kinetics and nucleic acid binding brought about by deletion of the conserved HBD and the adjacent domains suggest that the HBD contributes to RNA cleavage by promoting interaction with heteroduplex. In addition, we observed that the ablation of the HBD abolishes supershifting upon EMSA analysis, suggesting that two protein monomers may associate with a single substrate, conferring processivity to the enzyme, as previously suggested for the mammalian enzyme (23). Regions II and III initially attracted our attention, due to variability in length and composition among eukaryotes. In the *Drosophila* enzyme this extended linker region is particularly long (Fig. 1), with a predicted isoelectric point (pI = 5.18) similar to that of mammals or zebrafish, but lower than in *Xenopus tropicalis* (pI = 9.11) or *C.elegans* (pI = 6.79). Moreover, structural modelling suggested that Region III may constitute a second HBD (Supplementary Fig. S1C). We were unable to study its properties in isolation, due to its insolubility, possibly indicating that its folding depends on the adjacent domains. However, our other data (Tables II and III) indicate that, whereas this region actually weakens the overall hybrid binding of the enzyme (Fig. 3), it enhances catalysis (Table I). Although a conclusive interpretation of these findings must await full elucidation of the reaction mechanism of *Dm* RNase H1, it is tempting to suggest that the HBD-like fold of Region III is involved in shuttling substrate from the conserved HBD to the catalytic domain, as part of the processivity mechanism. Its deletion would thus promote tight and persistent hybrid binding by the conserved HBD, leading to a lower catalytic throughput. An alternative explanation for the findings might be that the length of the extended linker *per se* determines the catalytic activity and binding affinity of the enzyme. In other words, a long linker allows the catalytic domain to interact processively or successively with substrate, while also influencing binding (a ‘running dog on a leash’ model). Whether the ‘second HBD’ actually binds nucleic acid, even transiently, and whether its predicted fold is functionally important, must await detailed structural analysis and further mutagenic studies of the enzyme, using crystallography. The differences in the binding properties of the enzyme *in vitro*, using the linear hybrid (Fig. 2) and R-loop substrates (Fig. 3), may reflect functionally important differences *in vivo*, such as in the removal of persistent heteroduplex regions that arise during transcription, versus the processing of DNA replication intermediates (*e.g.* the formation and removal of leading- and lagging-strand primers).

The catalytic domain was predicted to adopt a similar fold as previously observed in viruses (61), bacteria (25, 26, 62, 63) and mammals (27), and includes the conserved DEDD motif (Supplementary Fig. S1), from which residue D252 was shown to be essential for activity, as in other organisms (34). Importantly, the catalytic domain was required for the 2:1 binding model (Table III), but not for the supershift seen in EMSA (Fig. 5A), for which the conserved HBD alone was sufficient. This suggests that the reaction involves not only the binding of a second protein molecule but also, potentially, a conformational change dependent on the catalytic domain, which may enable the second protein moiety to bind.

To test these ideas in the future, it may prove useful to create chimeric enzymes, for example inserting the second HBD from the *Drosophila* enzyme into the human one. However, since the activity of any domain of a protein is potentially influenced by any other, all possible chimeric constructs between the two enzymes should be tested, both with and without the additional HBD. This is particularly important for a processive enzyme like RNase H1, where each domain can be considered as one of its crucial moving parts.

### Functional comparison with RNase H1 from other species

In this study we adopted the same approach, using purified proteins, as has been successfully employed by others to study human RNase H1 and other enzymes of mitochondrial nucleic acid metabolism (64–66). Most studies of eukaryotic RNase H1 have focused on the enzymes from yeast and from humans, while little was known about the function, role and biochemical properties of the *Drosophila* enzyme (2, 27, 47). We initially characterized the enzymatic activity of *Dm* RNase H1 at 30°C, revealing slightly different kinetic parameters from those of the human enzyme, studied previously at 37°C (34, 45); 30°C has widely been used as a reference temperature for studies of *Drosophila* enzymes, notably those involved in mtDNA metabolism. It represents a temperature about 10–12°C warmer than the typical physiological temperature of the fly. However, assuming that, like their mammalian counterparts, *Drosophila* mitochondria are 10–12°C warmer than the cells and tissues in which they function (67), 30°C should represent an optimal temperature at which to study mitochondrially localized enzymes. When we compared the human and *Drosophila* enzymes we found their properties to be highly influenced by temperature, with the human enzyme essentially inactive at 30°C, but the *Drosophila* enzyme exhibiting a much lower substrate affinity (higher *K*_M_) at 37°C than at 30°C. At its presumed optimal temperature of 30°C, the *Drosophila* enzyme displayed a markedly higher affinity and catalytic turnover rate than the human enzyme at 37°C (Table I). However, given the marked temperature sensitivity of both enzymes, and the fact that the *in vivo* operating temperature of the human enzyme in mitochondria is probably much closer to 50°C than to 37°C (67), the two enzymes may have more similar properties than is apparent. Note also that the cell nucleus should be much closer to ambient temperature (in the fly) or to 37°C in humans, such that the enzymatic properties of RNase H1 in the nucleus may differ substantially.

Similar caution should apply to measurements of affinity constants, especially given the uncertainties raised by the use of different methods in the various studies. Here, applying BLI using the D252N-substituted enzyme, we inferred a *K*_D_ value intermediate between those previously reported for human (34) and *E. coli* RNase H1 (*67*). Previously, BLI has been used to measure nucleic acid–protein interactions, obtaining similar values as with other approaches (69), and has been specifically applied to the study of Polγ (53).

Mammalian, yeast and *E. coli* RNase H1 bind dsRNA, but only the enzyme from the archaeon *Sulfolobus tokodaii 7* has been demonstrated to digest this substrate (70). In the present study we found that *Dm* RNase H1 also binds dsRNA and dsDNA (Fig. 4 and Table II), respectively, ∼10-fold and ∼100-fold less tightly than RNA/DNA hybrid, but does not digest these substrates, nor does it bind or digest ssRNA or ssDNA, properties shared with the human and *E. coli* enzymes (64).

In a previous study, RNase H1 from the budding yeast *S. cerevisiae* was found to have two nucleic acid-binding domains located in the N-terminal region (*56*) (see Fig. 1),with the more N-terminally located such region showing a much higher affinity for substrate. This raises the question as to whether the architecture and suggested reaction mechanism of the *Dm* enzyme, with two HBDs, might also apply in yeast. However, in other respects the *Dm* enzyme differs fundamentally from that of *S. cerevisiae*, having much lower affinity for dsRNA than for RNA/DNA hybrid, while the ‘HBD’ of the yeast enzyme actually binds dsRNA more tightly than hybrid (57). Furthermore the catalytic domain of the *Dm* enzyme binds hybrid on its own, and is required for binding to dsRNA, while the enzyme from *S. cerevisiae* shares neither property (57). Thus, the functional properties of the two enzymes *in vivo* are likely to differ substantially.

### Significance of RNase H1 interactors

RNase H1 has been implicated in several processes in different sub-cellular compartments. Our mass spectrometry analysis of proteins associating with RNase H1 after cross-linking revealed a list of potential interactors in the nucleus, as well as in mitochondria, in both of which single-stranded DNA-binding proteins were prominent. In mammals, RPA (replication protein A) has been shown to recruit RNase H1 to R-loops and stimulate its enzymatic activity (24), via an interaction with the HBD. Such an interaction facilitates the role of the enzyme in heteroduplex surveillance and also, potentially, in DNA maintenance. SSB and RNase H have also been reported to interact in *E. coli* (71), in this case via the catalytic core, because the bacterial enzyme lacks the HBD. RPA (RpA-70) was one of the top nuclear hits in our screen for interacting proteins (Table IV), which also yielded two subunits of the nuclear replicative helicase, Mcm2 and Mcm3 (*71*, *72*), while RPA has been implicated in nuclear processes other than DNA replication (74, 75). We also identified mtSSB, the functional homologue of RPA in mitochondria, as a prominent hit (Table V). mtSSB is a well-characterized component of the mtDNA replication machinery (76), while RNase H1 in *Drosophila* has also been inferred to play a role in mtDNA replication fork progression (2). Furthermore, mtSSB and RNase H1 have been proposed to co-operate in the initiation of mtDNA replication (10), which spurred us to examine the possible interaction between them in more detail. The two proteins did not co-immunoprecipitate when overexpressed together *in vivo* (Fig. 6A and Supplementary Fig. S8), nor did enzymatic assays and EMSA reveal convincing evidence for any direct interaction *in vitro* (Fig. 6B–D), apart from a modest stimulation or possible inhibition of the enzyme, depending on the specific substrate (Fig. 6B and C). Although overexpression *in vivo* and *in vitro* analysis of the properties of bacterially expressed proteins is subject to different potential artefacts, the fact that neither approach strongly supported a direct and stable interaction implies that mtSSB and *Dm* RNase H1 most likely interact indirectly or transiently *in vivo*, requiring an unidentified partner protein or nucleic acid moiety to have enabled their co-detection by mass spectrometry. This does not exclude that the two proteins may co-operate as suggested (10), with mtSSB binding to the single-stranded DNA displaced at an R-loop, promoting RNase H1 recruitment that would partially digest the annealed RNA, thus creating a 3′-end accessible for extension by Polγ. However, it would imply that at least one additional partner would be required for such a recruitment to occur. This partner cannot simply be ssDNA, because it did not facilitate direct interaction between the proteins *in vitro* (Fig. 6B–D). It is also possible that mtSSB and RNase H1 co-localize at the replication origin, at replication forks and at dispersed R-loops only by virtue of their substrates (ssDNA and RNA/DNA hybrid) being juxtaposed at these sites, and thus that their close association is purely accidental. Importantly, negative findings such as ours, even though supported by multiple approaches, may be erroneous if the conditions for analysis *in vitro* are inappropriate. Future experiments using different methods may be needed to confirm (or revise) the apparent absence of direct interaction. The functional interactions of mtSSB with other mitochondrial replication proteins, such as Polγ (77) or mtDNA-helicase (78), require low salt conditions, similar to those used here, which yielded negative findings. However, it is possible that some other feature of the intramitochondrial environment is required to maintain mtSSB/RNase H1 links in *Drosophila*.

In human cells, mtSSB has been reported to localize partially to RNA granules (79), while defects in the machinery of RNA processing and degradation result in the accumulation of persistent R-loops, resulting in RNase H1 recruitment to nucleoids (80). In an earlier study, vertebrate RNase H1 was not observed as a nucleoid protein (81), consistent with its interactions with mitochondrial replication and RNA processing enzymes being transient, and mediated by its association with RNA/DNA hybrid substrate, rather than by direct protein–protein interactions.

Among other nuclear hits, we identified a second component of the R-loop processing machinery, the RNA helicase Rm62, the *Drosophila* homologue of human DDX5. In flies Rm62 is involved in transcriptional deactivation (82) and in the maintenance of chromatin states (83, 84), while human DDX5 functions in R-loop processing (85) and may perform a wide variety of additional role (86–88). The appearance of Rm62 in the list suggests a potential interaction between two independent machineries to resolve R-loops. The list of mitochondrial candidates was much longer, and included, as a prominent class, many metabolic enzymes involved in core processes such as fatty acid oxidation, the TCA cycle and OXPHOS, as well as some proteins involved in mitochondrial translation. Metabolic enzymes have been previously reported as at least peripheral components of nucleoids in many species, and there is on-going debate as to whether this association is meaningful. In regard to the present study, the question arises as to whether they represent ‘real’ interactors with RNase H1 or are just passenger proteins brought along by cross-linking in a protein-rich environment. Some hits, such as the fly orthologues of human TIMM44, GRPEL1, PITRM1 and PMPCA, are likely to be artefacts of overexpression, resulting from the machinery of protein import and processing becoming overwhelmed, even though these proteins did not appear in the mtYFP negative control list. Given the fact that the mitochondrial candidate list is ‘over-inclusive’ in this manner, and that many nuclear hits are congruent with previous data or with assumptions made on the basis of such data, the absence of any known component of the apparatus of mitochondrial nucleic acid metabolism other than mtSSB is striking.

Note that a number of other possible candidates do not figure in Tables IV and V because of the strict exclusion criteria, *i.e.* they were found in at least one control replicate (see Supplementary Table S2). Prominent among these was P32 (CG6459 in *Drosophila*), reported as being present in both the mitochondria and nucleus and implicated in diverse processes, and which was previously found to associate with RNase H1 in mammals and proposed to stimulate its activity (45). While it may be enriched in the fraction associating with RNase H1, it is not specific to this fraction.

Having undertaken this study to follow up previous observations that a deficiency of *Dm* RNase H1 results in characteristic abnormalities of mtDNA replication and transcription (2), the absence of replication and transcription proteins other than mtSSB from the list of mitochondrial positives was unexpected. Furthermore, the clinical features manifested by patients with mutations in the *RNASEH1* gene resemble those associated with other disorders caused by defects in the mtDNA replication apparatus (37–39). One simple explanation is that RNase H1 does not interact directly with replication proteins, and that the effects of its deficiency on mtDNA replication are secondary to a failure to process R-loops and other hybrid-containing structures. In other words, RNase H1 may function independently and not be part of the mitochondrial replisome, transcriptional machinery or any other protein complex within or associated with the nucleoid.

## Supplementary Data

Supplementary Data are available at *JB* Online.

## Supplementary Material

mvaa067_supplementary_data

## Abbreviations

BLI: biolayer interferometry
Dm: *Drosophila melanogaster*
EMSA: electrophoretic mobility shift assay
GMQE: global model quality estimation
HBD: hybrid-binding domain
LB: L-broth
mtDNA: mitochondrial DNA
PDB: protein data bank
RNase H1: ribonuclease H1
*rnh1*: ribonuclease H1 (*Drosophila* gene name)
RPA: replication protein
TFAM: mitochondrial transcription factor A
TBE: Tris-borate-EDTA buffer
YFP: yellow fluorescent protein.
