# Supplementary material for: A second hybrid-binding domain modulates the activity of Drosophila ribonuclease H1
Source: J Biochem. 2020 Jul 2;168(5):515–33. doi: 10.1093/jb/mvaa067 (PMC7657459; doi:10.1093/jb/mvaa067)
Supplement: mvaa067_supplementary_data [file mvaa067_supplementary_data.pdf]

A second hybrid-binding domain modulates the activity of *Drosophila* ribonuclease H1

Jose M. González de Cózar, Maria Carretero-Junquera, Grzegorz L. Ciesielski,

Sini M. Miettinen[3], Markku Varjosalo, Laurie S. Kaguni,

Eric Dufour[1] & Howard T. Jacobs

SUPPLEMENTARY DATA

Contents

Supplementary Table S1

Legends to Supplementary Figures (S1-S8)

Supplementary Figures S1-S8

Please note: Supplementary Table S2 is available as a separate Excel file, Table S2.xls

SUPPLEMENTARY TABLE S1

Oligonucleotides used in the study

**Oligonucleotide Name**	**Sequence (5'-3')[a]**	**Purpose**
30nt_RNA	CGCGAAGACUAACUAUAGCGUGCCCUACUU	Nuclease activity and binding affinity test
30nt_RNA_C	AAGUAGGGCACGCUAUAGUUAGUCUUCGCG	Nuclease activity and binding affinity test
30nt_DNA	AAGTAGGGCACGCTATAGTTAGTCTTCGCG	Nuclease activity and binding affinity test
30nt_DNA_C	CGCGAAGACTAACTATAGCGTGCCCTACTT	Nuclease activity and binding affinity test
60nt_ssDNA	TCATCAGTAGAAGAGACCATGAGGGCTCCCTGACAGATCCCTATGTCGCTTCCGCCGTCA	ssDNA for complexing with mtSSB
90nt_Rloop_30_1	TGACGGCGGAAGCGACATAGGGATCTGGGGATGAAGCAATCCAACGCTACGCA TGTGAAGTACAGCCCTCATGGTCTCTTCTACTGATGA	Nuclease activity and binding affinity test
90nt_ Rloop_30_2	TCATCAGTAGAAGAGACCATGAGGGCTCCCAAGTAGGGCACGCTATAGTTAGTC TTCGCGTGACAGATCCCTATGTCGCTTCCGCCGTCA	Nuclease activity and binding affinity test
Biotinylated_30nt_RNA	AAGUAGGGCACGCUAUAGUUAGUCUUCGCG	Biolayer interferometry probe
Biotinylated_30nt_DNA	AAGTAGGGCACGCTATAGTTAGTCTTCGCG	Biolayer interferometry probe
RNaseH1_pET26b_F	AAACATATGGCGTTTTACGCTGTAGCC	Cloning *rnh1* coding sequence (commencing at Met-16) into pET26b and removing the stop codon, allowing readthrough into the His tag of the vector
RNaseH1_pET26b_R	TTT*CTCGAG*ACCATTTTTCTGCTTATACAAGGC	
RNaseH1_D252N_Fw	TGCATCAGCACAAACTCTCAGTTTTTG	Generation of inactive RNase H1 variant

RNaseH1_D252N_Rv	CCACAGCGTTATGGAGTTGAT	Generation of inactive RNase H1 variant
RNaseH1_P_87Fw	AGACCTCATATGAGTATCGAAGTGAATAAGAAC	Generation of ΔI/II variant, used with RNaseH1_pET26b_R
RNaseH1_P65L_Rv	CGTGCCGCAGCCATTAACGAACTGATC	Generation of ΔII/III
RNaseH1_142L_Fw	GGCTGCGGCACGACCAGCGGCGATAAG	
RNaseH1_P142_Fw	AAA*CATATG*GGCACGACCAGCGGCGATAAG	Generation of ΔI-III, used with RNaseH1_pET26b_R
RNaseH1_P179_Fw	AGACCT*CATATG*GGCTATGTCATTGTGTACACA	Generation of ΔI-IV, used with RNaseH1_pET26b_R
RNaseH1_87L_Rv	GTTTTTCCAACTGGCCAGTGA	Generation of ΔIII/IV by blunt-end ligation
RNaseH1_179L_Rv	GGCTATGTCATTGTGTACACA	
RNaseH1_P87_Rv	CTCGG*CTCGAG*CCAACTGGCCAGTGACGCCTT	Generation of ΔIII-V, used with RNaseH1_pET26b_F
RNaseH1_P65_Rv	CTCGG*CTCGAG*CGACTTGCAGCCATTAACGAA	Generation of ΔII-V, used with RNaseH1_pET26b_F
RNaseH1_P142_Rv	CTCGG*CTCGAG*GCCCTTCCGCTTGCGATTGAG	Generation of ΔIV/V, used with RNaseH1_pET26b_F and of ΔI/II-ΔIV/V, used with RNaseH1_P_87Fw
pMT_mtSSB_Fw	GTGGTG*GAATTC*AAGATGCAACACACAAGGCGCAT	Cloning of *mtSSB* coding sequence for in-frame fusion to C-terminal HA tag
pMT_mtSSB_Rv	TCTAGA*CTCGAG*GTTGTTGGCATCACGGAAAAAC	

KpnI_mtSSB HA_PmeI forward	AGATCGG*GGTACC*TACTAGTCCAGTGTGGTGGAA	Recloning of mtSSB into pMT-puro, removing V5/His tag and inserting HA tag
KpnI_mtSSB HA_PmeI reverse	CAGCGG*GTTTAAAC*TCAAGCGTAATCTGGAACATCGTATGGGTATTCGAAGG GCCCTCTAGACTCGAGGTTGTTGGCATC	

[a]introduced restriction sites denoted by italics, mutated nucleotide (GAC>AAC, Asp>Asn) denoted by underline

LEGENDS TO SUPPLEMENTARY FIGURES

Figure S1

Structure prediction proposes an additional HBD located within the 'linker region' of *Dm* RNase H1

Protein sequence comparison of *Drosophila* and human RNase H1, the template used for building the model. The protein sequence is divided into three regions: (A) conserved HBD (*Dm*, shown as amino acids 15-66), (B) second predicted HBD (*Dm*, shown as amino acids 85-142) and (D) RNase H catalytic domain (*Dm*, shown as amino acids 171-333). Amino acids involved in nucleic acid recognition are marked as #; those involved in RNA catalysis are marked as &. Note that the structure of the human enzyme was derived from a variant bearing the D210N substitution, producing a catalytically inactive enzyme for crystallography. *Hs* amino acid D210 is equivalent to D252 in the *Dm* enzyme. (C) Comparison of protein structure of *Hs* HBD (DOI: 10.2210/pdb3bsu/pdb) with predicted structures of the two proposed HBDs of the *Dm* protein. Center panel represents the *Dm* conserved HBD (amino acids 17-64) and right panel represents the second, predicted HBD (amino acids 87-141). (E) Comparison of protein structure of human RNase H catalytic domain (DOI: 10.2210/pdb4H8K/pdb) to predicted *Dm* RNase H catalytic domain (amino acids 175-329). (F) Catalytic core of *Hs* (left) and *Dm* (right) RNase H domain, indicating the residues of the conserved DEDD motif.

Figure S2

Original data for extrapolating properties of RNase H1 and variants

(A, B, C) Kinetic parameters. In 10 µl reactions, 2.5, 5, 7.5 and 10 nM (i.e. 25-100 fmol) of a 30 bp 5´-radiolabeled RNA/DNA hybrid was incubated with 2 fmol of human (Hs) or *Drosophila* (Dm) RNase H1 or the indicated variants, for times and at temperatures as shown, followed by separation on non-denaturing gels. Initial cleavage rates (V_0) were plotted for each protein, incubated at (B) 37 ºC or (C) 30 ºC according each initial substrate concentration (S_0), based on densitometric analysis of the gels. All plotted values are means ± SD of three replicate experiments. (D) Binding properties, based on EMSA, using the D252N variant of *Dm* RNase H1, and RNA/DNA hybrids of different lengths as indicated, at the protein molar excess levels shown. Putative monomeric and dimeric complexes are indicated by the open and filled

arrowheads, respectively. In this and subsequent figures RNA and DNA are represented by red and black bars, respectively.

Figure S3

Region I (conserved HBD) is required for dsDNA binding

BLI analysis of binding of RNase H1 and variants, each carrying the D252N substitution, to 30 bp dsDNA. Streptavidin sensors were incubated in 80 µl of 25 nM 5´ biotinylated dsDNA solution. Association was measured by transferring sensors to 80 µl of different concentrations of protein solution (0, 100, 200, 400, 600, 800 and 1000 nM). The sensogram displays the baseline, association and dissociation steps, with experimental data shown as black lines and, where binding was detected, the extrapolated data fitted to a heterogeneous (2:1) binding model shown as red lines. See Table 2 for association/dissociation parameters.

Figure S4

Region I (conserved HBD) is required for dsRNA binding

BLI analysis of binding of RNase H1 and variants, each carrying the D252N substitution, to 30 bp dsRNA. Streptavidin sensors were incubated in 80 µl of 25 nM 5´ biotinylated dsDNA solution. Other details as for Fig. S2.

Figure S5

RNase H1 lacks ssDNA or ssRNA binding capacity

EMSA, using 2 pmol of a 30 nt 5´-radiolabeled (A) ssRNA or (B) ssDNA, incubated with different amounts of RNase H1 and variants (1 or 10 µM), each carrying the D252N substitution. Reaction products were separated by non-denaturating electrophoresis. In this and subsequent figures RNA and DNA are represented by red and black bars, respectively, with radiolabel indicated by the asterisk.

Figure S6

Regions IV + V are required for dsDNA or dsRNA binding

BLI analysis of further RNase H1 variants lacking domains IV+V as indicated, using (A) dsDNA or (B) dsRNA as substrate. Other details as for Fig. S4A. No binding was observed to any of these variant proteins.

Figure S7

mtYFP and mtSSB are targeted to mitochondria

Representative images from immunocytochemistry of cells transiently transfected with (A) pMT-mtYFP and (B) pMTpuro-mtSSB-HA, probed for (HA tag or mtYFP, green), cytochrome oxidase subunit 4 (COXIV, red), and DAPI (blue). Scale bars 20 μm.

Figure S8

Repeat of co-immunoprecipitation test (Fig. 6A)

(A) Western blots of immonoprecipitates from S2 cells co-expressing RNase H1-V5 and mtSSB-HA, probed as indicated. Immunoprecipitates were tracked by successive samplings during the procedure, indicated as follows (bc – before crosslinking, ac – after crosslinking, p – pellet, ft – flowthrough, w1, 2 and 3 – washes, e – eluate, e+ – eluate after heat treatment to remove any persisting cross-links). Samples imaged using anti-HA to detect mtSSB-HA, anti-6x-His tag for RNase H1-V5/His and GFP for detecting mtYFP.

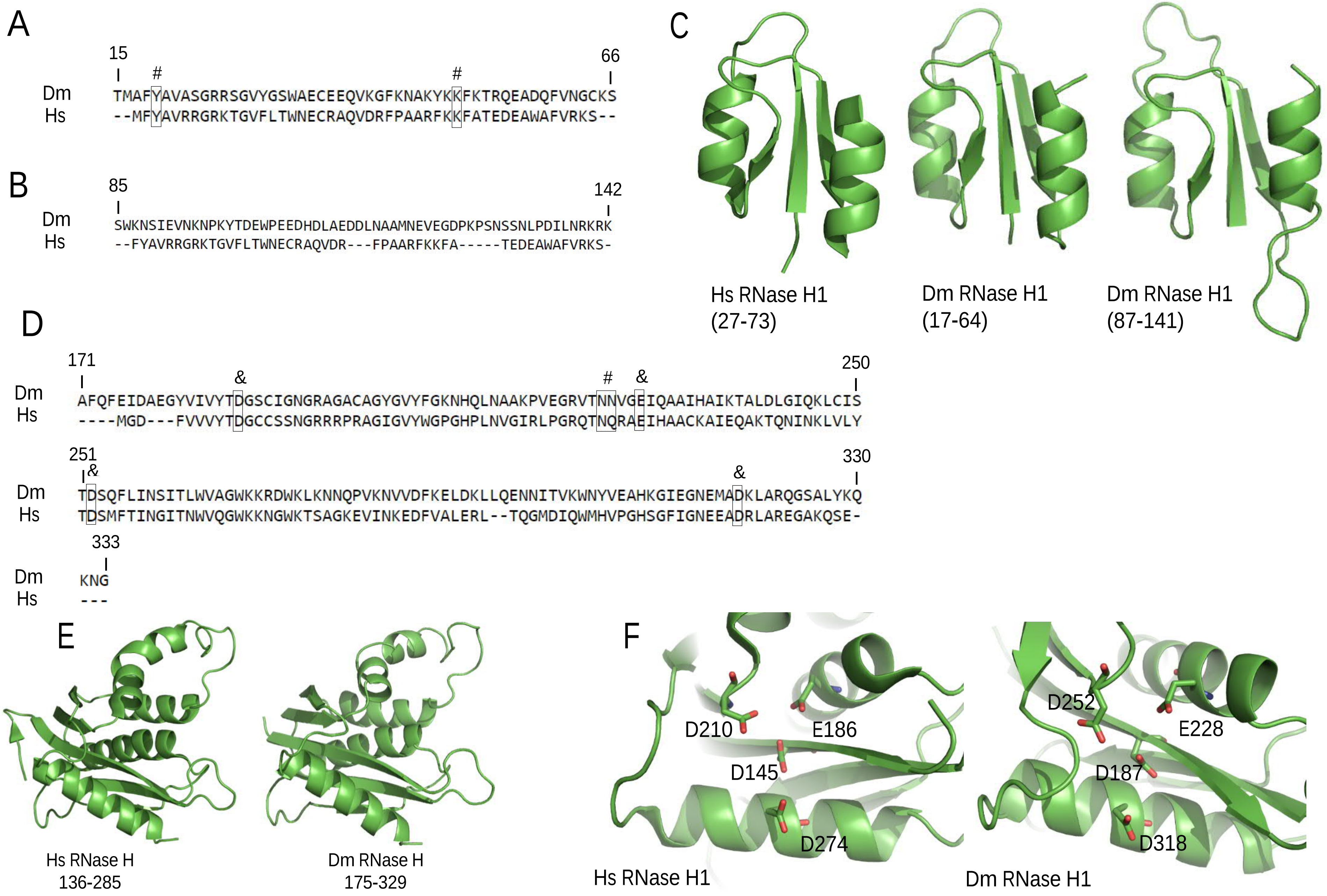

González de Cózar et al, Figure S1

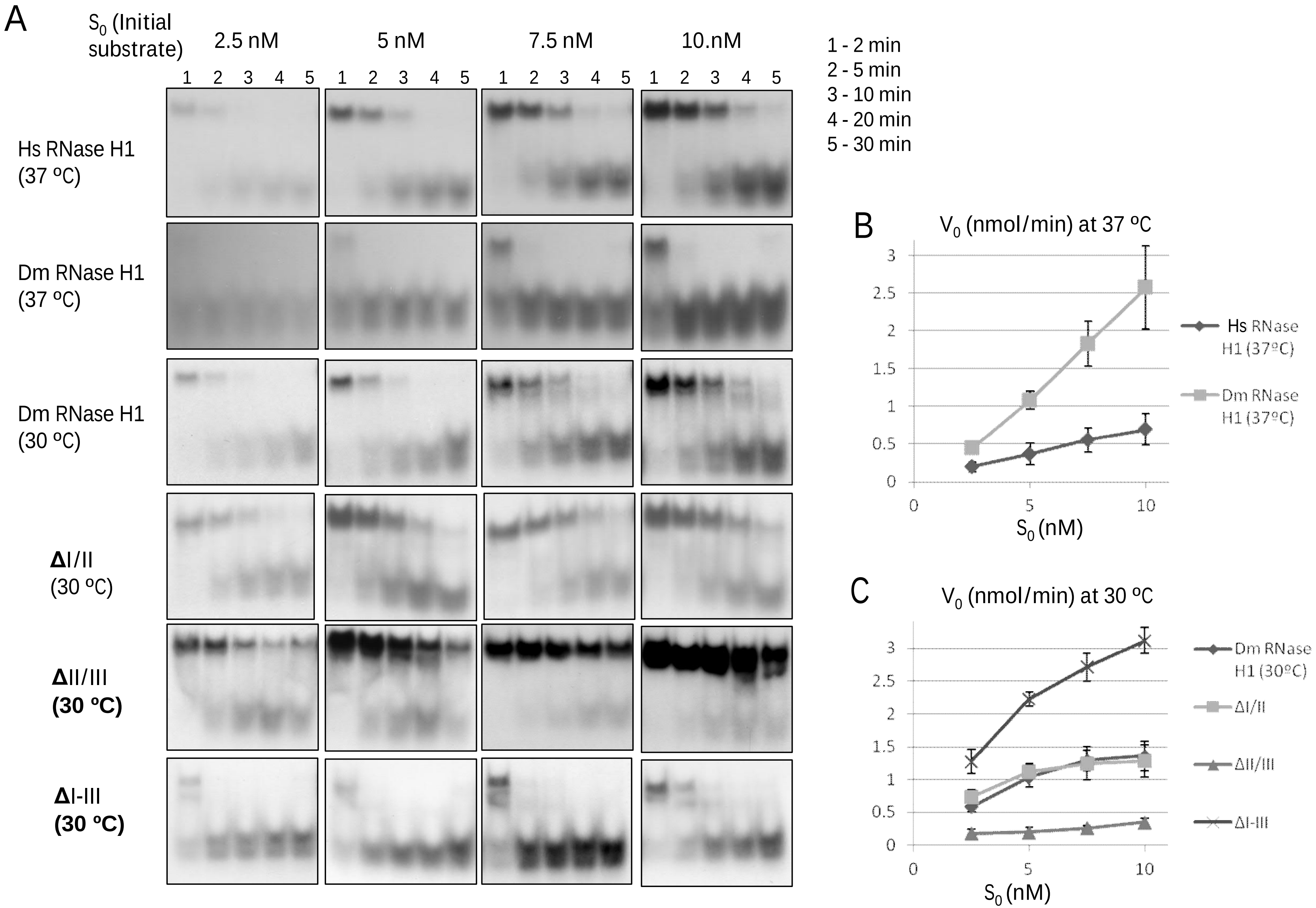
A
S0 (Initial substrate)
2.5 nM
5 nM
7.5 nM
10.nM
1 2 3 4 5
1 - 2 min
2 - 5 min
3 - 10 min
4 - 20 min
5 - 30 min
Hs RNase H1 (37 ºC)
Dm RNase H1 (37 ºC)
Dm RNase H1 (30 ºC)
ΔI/II (30 ºC)
ΔII/III (30 ºC)
ΔI-III (30 ºC)
B
V0 (nmol/min) at 37 ºC
Hs RNase H1 (37ºC)
Dm RNase H1 (37ºC)
S0 (nM)
C
V0 (nmol/min) at 30 ºC
Dm RNase H1 (30ºC)
ΔI/II
ΔII/III
ΔI-III
S0 (nM)

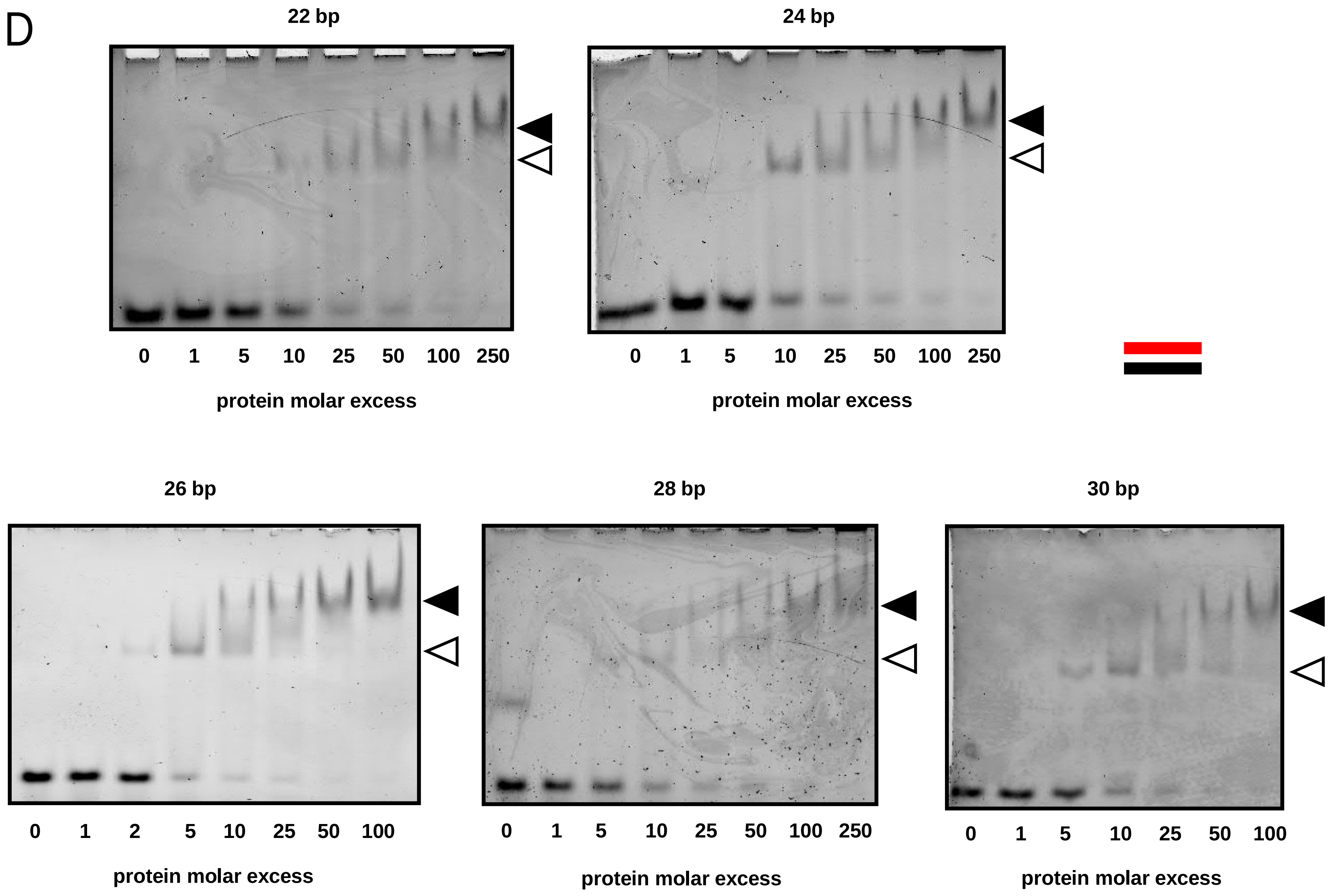
D
22 bp
0 1 5 10 25 50 100 250
protein molar excess
24 bp
0 1 5 10 25 50 100 250
protein molar excess
26 bp
0 1 2 5 10 25 50 100
protein molar excess
28 bp
0 1 5 10 25 50 100 250
protein molar excess
30 bp
0 1 5 10 25 50 100
protein molar excess

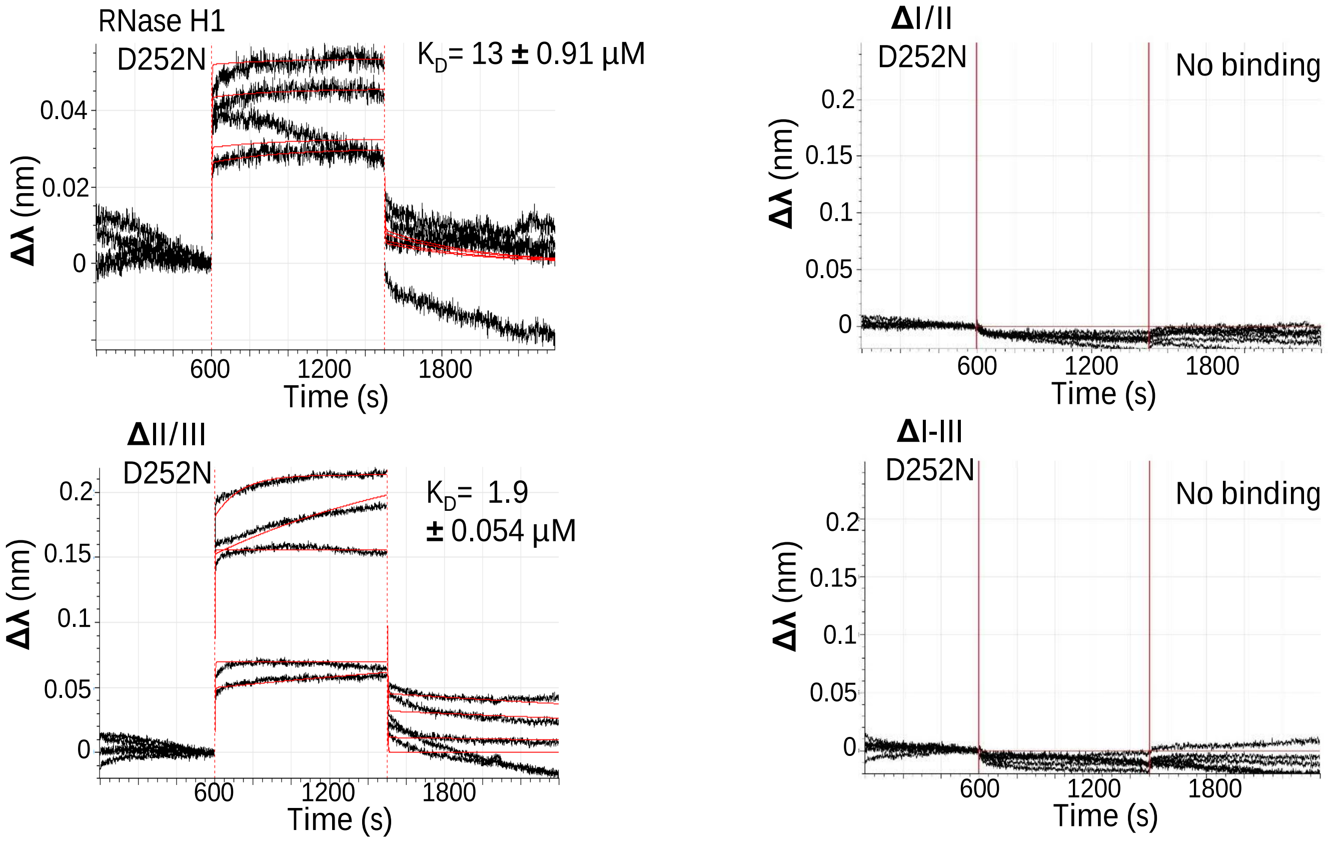

González de Cózar et al, Figure S3

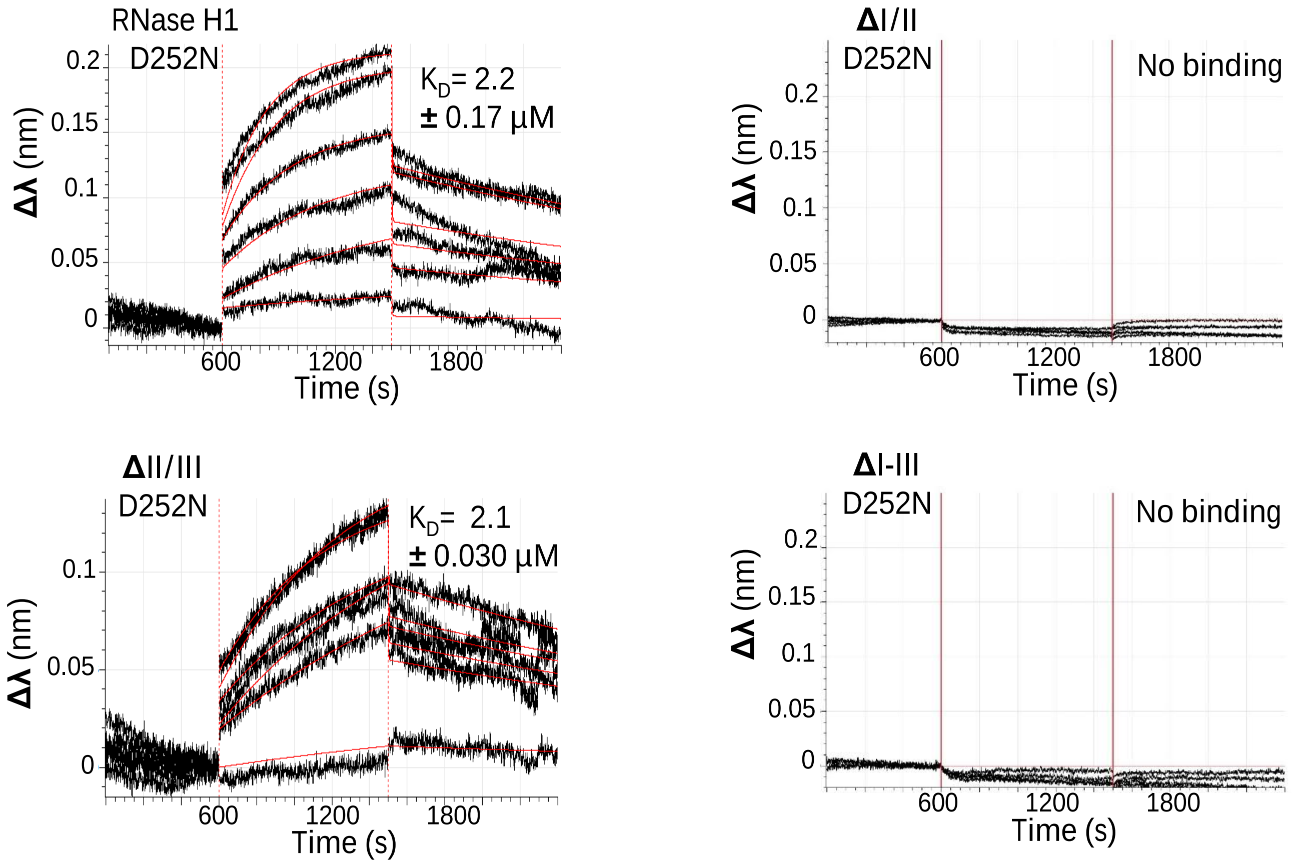

González de Cózar et al, Figure S4

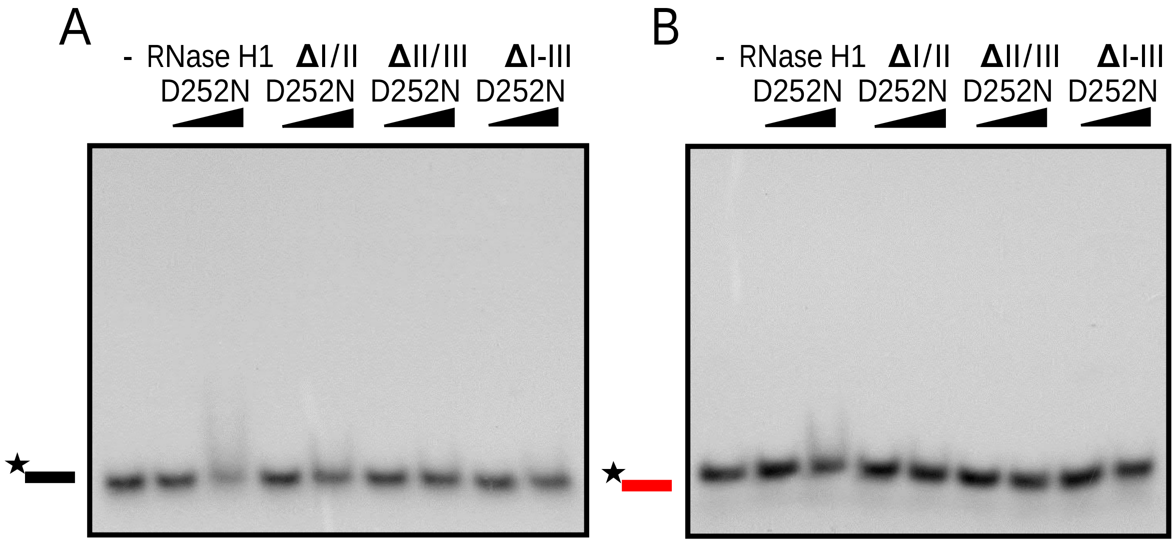

González de Cózar et al, Figure S5

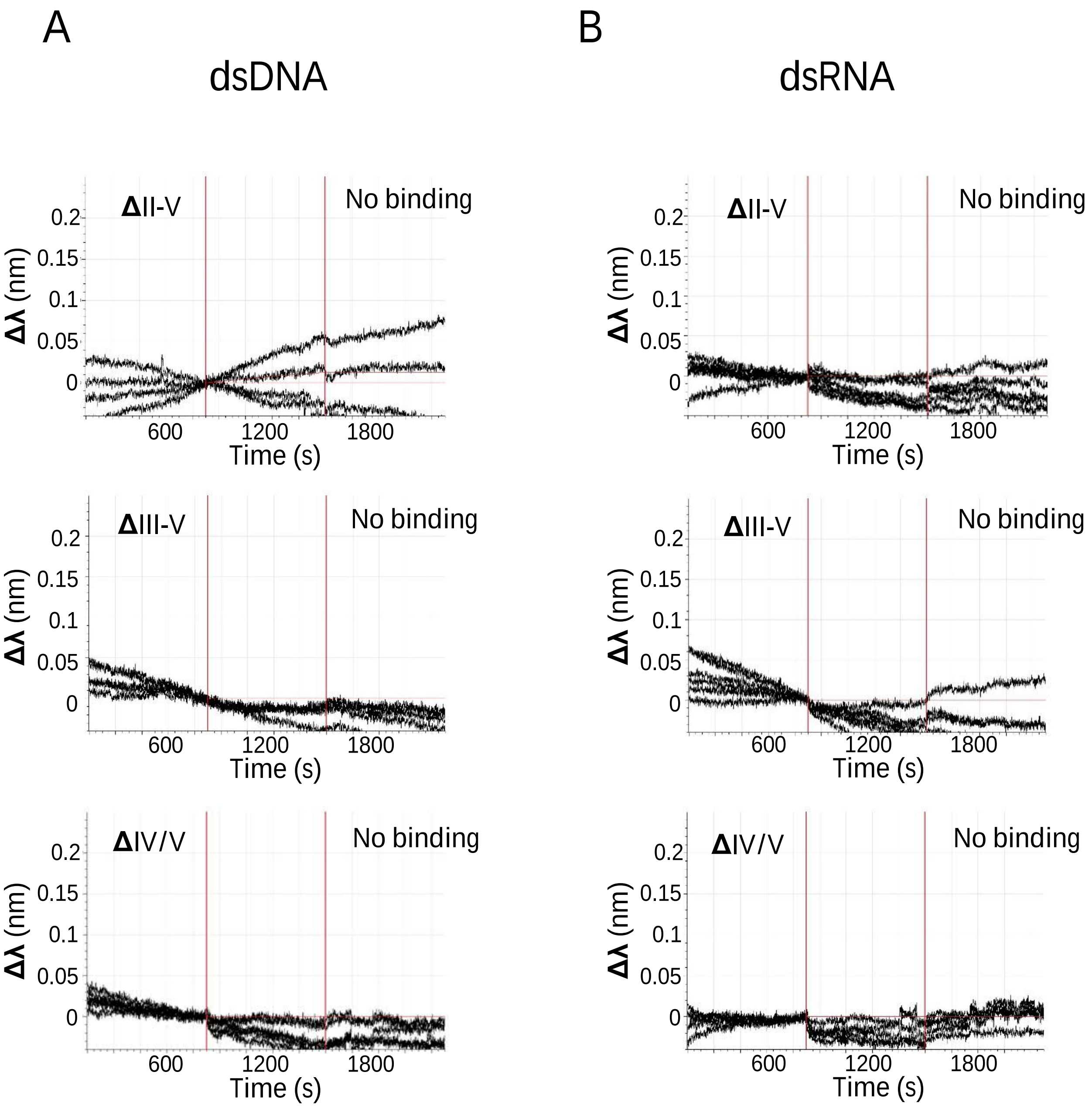

González de Cózar et al, Figure S6

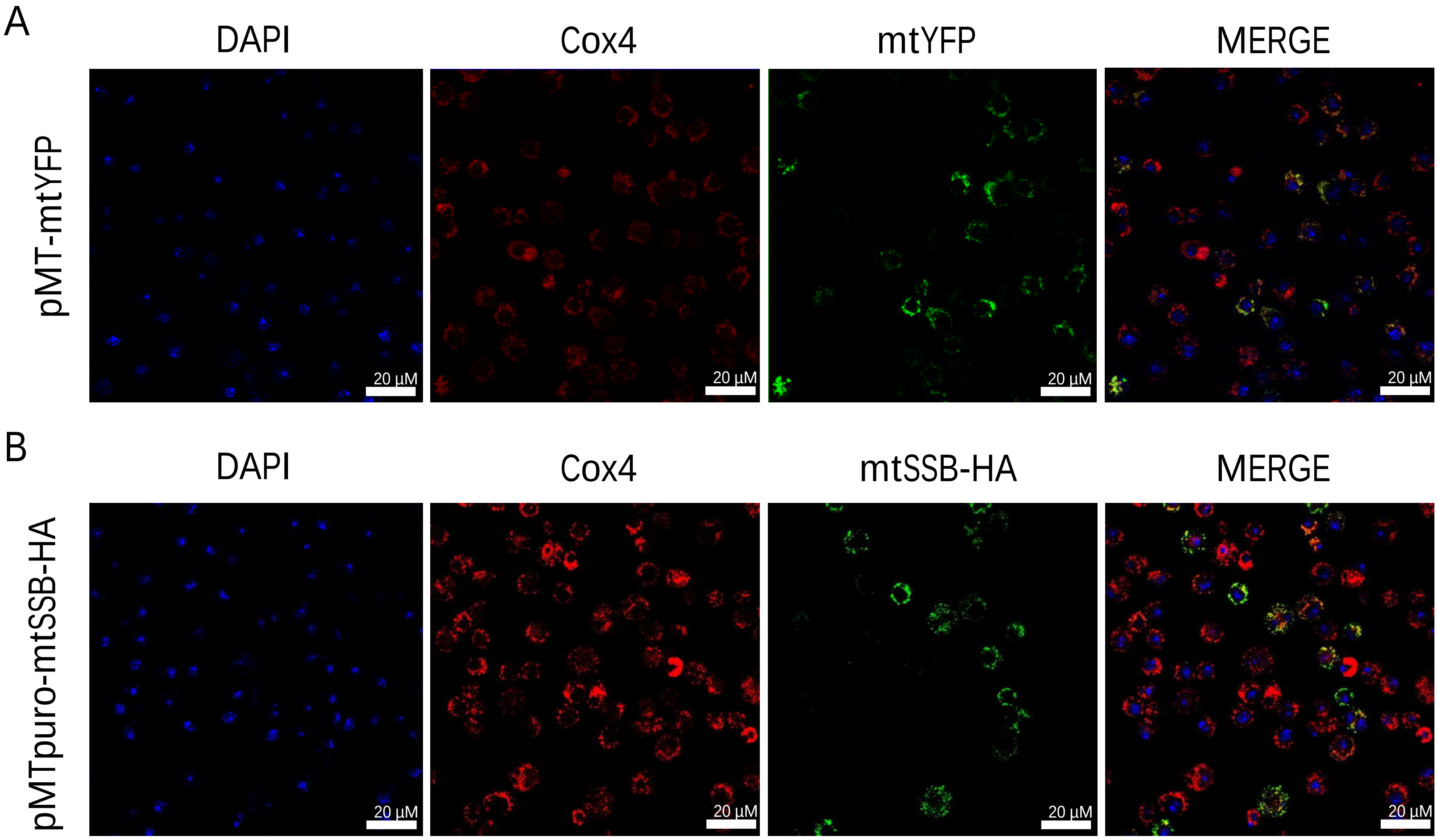

González de Cózar et al, Figure S7

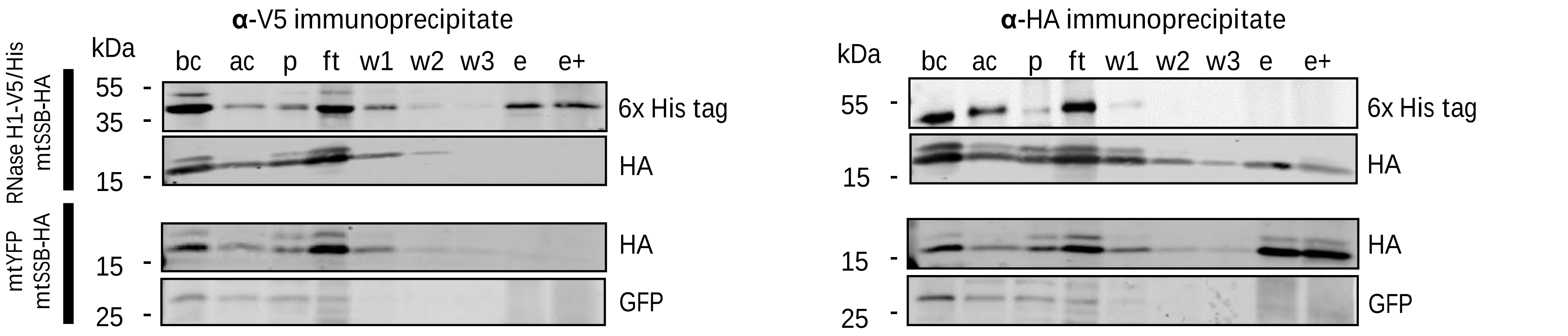

González de Cózar et al, Figure S8
